# The small GTPase, nucleolar GTP-binding protein 1 (NOG1), has a novel role in plant innate immunity

**DOI:** 10.1038/s41598-017-08932-9

**Published:** 2017-08-23

**Authors:** Seonghee Lee, Muthappa Senthil-Kumar, Miyoung Kang, Clemencia M. Rojas, Yuhong Tang, Sunhee Oh, Swarup Roy Choudhury, Hee-Kyung Lee, Yasuhiro Ishiga, Randy D. Allen, Sona Pandey, Kirankumar S. Mysore

**Affiliations:** 1 Noble Research Institute, LLC, Ardmore, Oklahoma USA; 2 Gulf Coast Research and Education Center, Institute of Food and Agricultural Science, University of Florida, Balm, Florida USA; 3 Institute for Agricultural Biosciences, Oklahoma State University, Ardmore, Oklahoma USA; 4 Donald Danforth Plant Science Center, St. Louis, Missouri USA; 5 Department of Plant Pathology, Present Address: University of Arkansas, Fayetteville, Arkansas USA; 6 Present Address: National Institute of Plant Genome Research, Aruna Asaf Ali Marg, New Delhi, India; 7 Department of Plant Pathology, Present Address: University of Tsukuba, Ibaraki, Japan

## Abstract

Plant defense responses at stomata and apoplast are the most important early events during plant-bacteria interactions. The key components for the signaling of stomatal defense and nonhost resistance have not been fully characterized. Here we report the newly identified small GTPase, *Nucleolar GTP-binding protein 1* (*NOG1*), functions for plant immunity against bacterial pathogens. Virus-induced gene silencing of *NOG1* compromised nonhost resistance in *N. benthamiana* and tomato. Comparative genomic analysis showed that two *NOG1* copies are present in all known plant species: *NOG1-1* and *NOG1-2*. Gene downregulation and overexpression studies of *NOG1-1* and *NOG1-*2 in Arabidopsis revealed the novel function of these genes in nonhost resistance and stomatal defense against bacterial pathogens, respectively. Specially, *NOG1-2* regulates guard cell signaling in response to biotic and abiotic stimuli through jasmonic acid (JA)- and abscisic acid (ABA)-mediated pathways. The results here provide valuable information on the new functional role of small GTPase, NOG1, in guard cell signaling and early plant defense in response to bacterial pathogens.

## Introduction

Plant pathogens that are able to cause disease in a given plant species are considered host pathogens while those that are unable to do so are nonhost pathogens. Nonhost resistance is a more wide-spread and durable plant defense mechanism that is achieved by a combination of preformed and inducible defenses^1, 2^. Preventing the entry of the pathogen into plant tissue is one of the key aspects of nonhost resistance, also known as stomatal innate immunity^3–6^.

In contrast to many fungal pathogens that are able to penetrate the plant epidermis, bacterial pathogens rely on wounds or natural openings to enter the apoplast^7, 8^. One well-characterized means of entry is through the stomata, microscopic pores on the plant surface that allow gas exchange between plant tissues and the atmosphere. Stomatal opening and closure depend on the environmental and physiological conditions of the plant and are regulated by two guard cells that surround the pore^9^. Pathogen Associated Molecular Patterns (PAMPs) such as flagellin-derived peptide flg22 and the bacterial lipopolysaccharide (LPS) can trigger stomatal closure^7^. However, adapted plant bacterial pathogens are able to re-open stomata by means of virulence factors such as the phytotoxin coronatine (COR), a mimic of the active JA-Ile hormone^4, 7^. In the absence of COR, transcription factors related to JA signaling such as MYC2 interact with a repressor complex formed by Jasmonate-Zim domain (JAZ) to repress transcription of JA-responsive genes^10^. In the presence of COR, JAZ proteins bind the F-box protein Coronatine insensitive 1 (COI1), a subunit of an E3 ubiquitin ligase complex, and are subjected to 26 S proteasome-mediated degradation^11^. Although JA regulated genes play a critical role in JA-mediated guard cell signaling pathway and stomatal immunity, it still remains unclear what genetic components are directly implicated in this sophisticated network that regulate stomatal defense against bacterial pathogens. In the present study, we identified two small G-proteins, *Nucleolar GTP-binding protein 1-1* (*NOG1-1*) and *1-2* (*NOG1-2*), which play an important role in the regulation of nonhost resistance and stomatal defense against bacterial pathogens.

G-proteins are GTP-binding proteins with GTPase activity that act as molecular switches to regulate diverse cellular processes by alternating between an active conformation (GTP-bound) and an inactive conformation (GDP-bound)^12^. Small monomeric G-proteins, also known as small GTPases, are widely conserved in eukaryotes and regulate many essential cellular processes^13^. Based on their function, these small GTPases are classified as: ADP ribosylation factor (ARF), Ras-related in brain (RAB), Ras-related nuclear protein (RAN), rat sarcoma (RAS) and RHO^14^. A number of RHO family of small GTPases are well known as the key regulator of immunity in plants and animals^15^. Some of the small RAB GTPases have been described for their important role for SA- and JA-mediated defense signaling, and stomatal immunity^16–18^. On the other hand, the family of TRAFAC (translation factors) belonging to the superclass of P-loop GTPases is a novel group of G-proteins, initially identified by analyzing fully sequenced bacterial genomes and essential for cell viability, and distinct from the small GTPases commonly present in plants^19–21^. The TRAFAC class is divided into seven families: TrmE (Probable tRNA modification GTPase in *E. coli*), Era (*E. coli* Ras-like protein), YfgK, YihA, OBG, Hflx and classic translation factor family^19^. The orthologs of these organelle-targeted small GTPases were found in plants, suggesting the horizontal transfer of eubacteria-derived small GTPases into plants. Most of them are localized to chloroplasts and/or mitochondria, while only the archaea-related GTPases in OBG and ERA family are localized to the cytoplasm and nucleus. In Arabidopsis, 18 GTPases belonging to the TRAFAC class have been identified^20^. The Arabidopsis NOG1-1 and NOG1-2 belongs to the OBG family that has been known to regulate ribosome biogenesis and RNA modification in yeast and bacteria. Recently, subcellular localization studies showed that NOG1 homologs in Arabidopsis were localized to the nucleus^22^. However, their biological functions are largely unknown in plants^23–25^. In this study, we showed that *NOG1-1* and *NOG1-2* are involved in plant defense against bacterial pathogens.

## Results

### *NOG1* is involved in nonhost resistance in *Nicotiana benthamiana* and tomato

**Figure 1:**
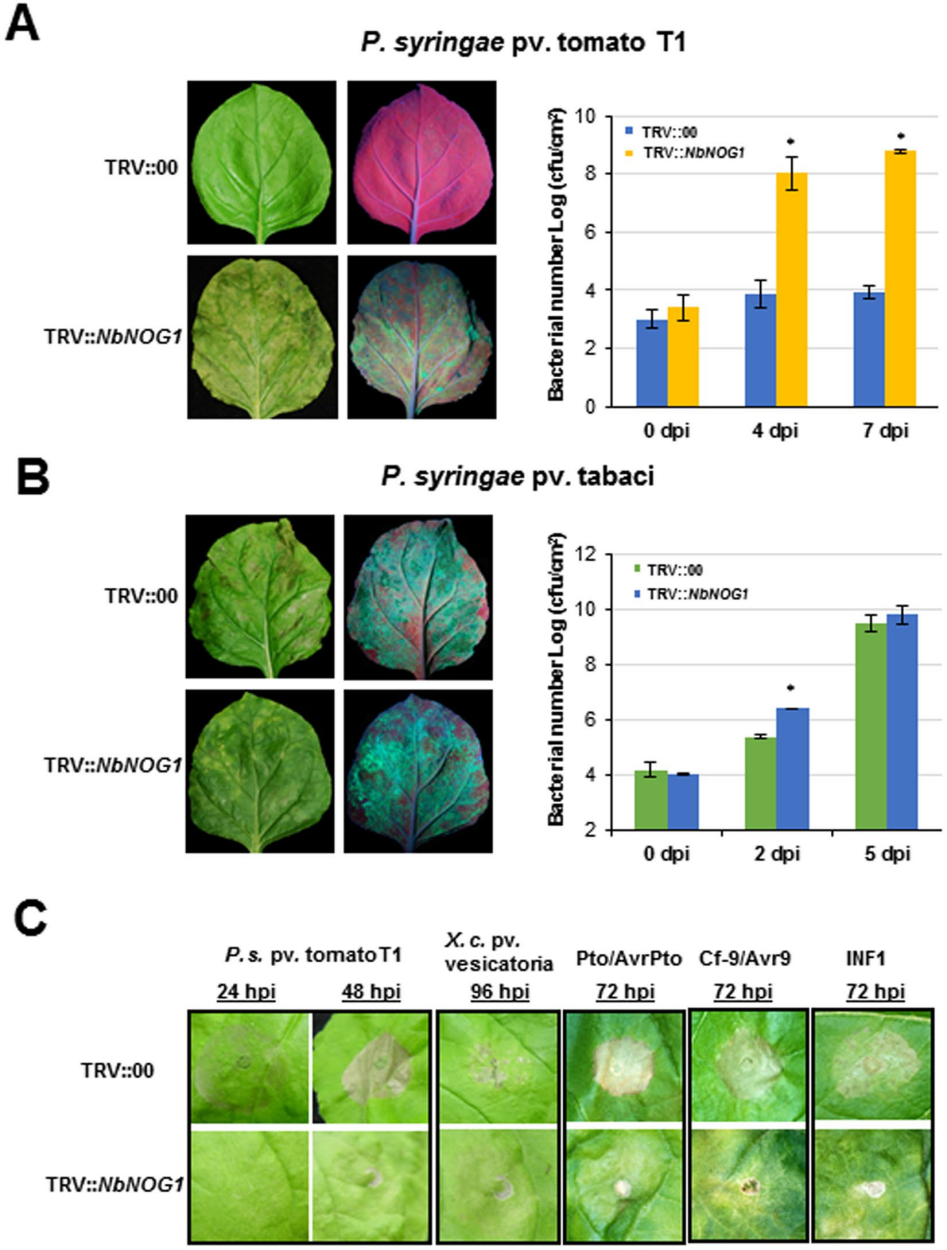
*N. benthamiana NbNOG1*-silenced plants are compromised in nonhost resistance. (**A**,**B**) *NbNOG1-*silenced (TRV::*NbNOG1*) and non-silenced control (TRV::00) *N. benthamiana* plants were vacuum-infiltrated with nonhost pathogen *P. syringae* pv. tomato T1 (*pDSK-GFP*_*uv*_) or host pathogen *P. syringae* pv. tabaci (*pDSK-GFP*_*uv*_) to observe symptom development (left panels) or bacterial multiplication 3 days post-inoculation (dpi; right panels). An increase in GFP fluorescence associated with bacterial multiplication was observed in TRV::*NbNOG1* plants but not in the TRV::00. To monitor bacterial multiplication in TRV::*NbNOG1* and TRV::00, *N. benthamiana* plants were vacuum-infiltrated with *P. syringae* pv. tomato T1 (**A**) and *P. syringae* pv. tabaci (**B**) and bacterial multiplication was quantified at various dpi as indicated. Bars represent means and standard deviations for three independent experiments. Asterisks above bars indicate statistically significant difference between *NbNOG1-*silenced plants and control (Student’s *t*-test *P < *0.05). (**C**) HR was observed between *NbNOG1-*silenced and control *N. benthamiana* plants. Plants were syringe-infiltrated with *P. syringae* pv. tomato T1 or *X. campestris* pv. vesicatoria (1 × 10^6^ CFU/ml) or *Agrobacterium* strains for transient expression of *Pto* and *AvrPto*, or *Cf-9* and *Avr-9*, or *INF1*. *Agrobacterium* strain GV2260 with empty vector (EV) was used as a control. HR was observed at different hours post inoculation (hpi). This experiment was repeated at least three times and showed similar results. Each experiment had five replications.

A *Tobacco rattle virus* (TRV)-based virus-induced gene silencing (VIGS)-mediated fast forward genetics approach was used in *N. benthamiana* to identify plant genes that play a role in nonhost resistance against bacterial pathogens^26^. One of the cDNAs identified from this approach had homology to the functionally uncharacterized gene with a small GTPase domain, *NOG1*. Upon inoculation with the nonhost pathogen *Pseudomonas syringae* pv. tomato T1, bacterial multiplication was significantly increased (>4 logs) in the inoculated leaves when compared to the non-silenced control (TRV::00) that was asymptomatic (Fig. 1A).

To assess how broad the *NOG1*-mediated nonhost resistance was, *NbNOG1*-silenced *N. benthamiana* plants were further analyzed for their response to additional nonhost pathogens such as *P*. *syringae* pv. glycinea (a soybean pathogen) and *Xanthomonas campestris* pv. vesicatoria (a pepper pathogen). The down-regulation of *NbNOG1* was confirmed in *NbNOG1*-silenced *N. benthamiana* plants (Fig. S1A). Both pathogens multiplied to significantly higher levels at 7 days post-inoculation (dpi) in *NOG1-*silenced plants compared to wild-type and non-silenced control plants (Fig. S1B,C). Inoculation with the host pathogen *P. syringae* pv. tabaci caused disease symptoms and bacterial multiplication in both *NbNOG1*-silenced plants and non-silenced controls with no significant difference at 5 dpi although more number of bacteria were found in infected leaves at 2 dpi (Fig. 1B).

To determine whether downregulation of *NOG1* impairs elicitation of the hypersensitive response (HR), a visual inspection of HR symptom development was performed in *NbNOG1-*silenced and control plants after infiltration with high inoculum of the nonhost pathogens *P. syringae* pv. tomato T1 and *X. campestris* pv. vesicatoria, or by transient co-expression of the resistance (*R*) genes *Pto* or *Cf9* with their corresponding avirulence genes *AvrPto* or *AvrCf9*, respectively, or by transient expression of the PAMP elicitor *INF1*. HR symptoms were observed in the control plants but not in the *NbNOG1-*silenced plants at the time points tested (Fig. 1C), suggesting that *NOG1* also plays a role in elicitation of the HR triggered by nonhost pathogens, gene-for-gene interactions and PAMPs.

**Figure 2:**
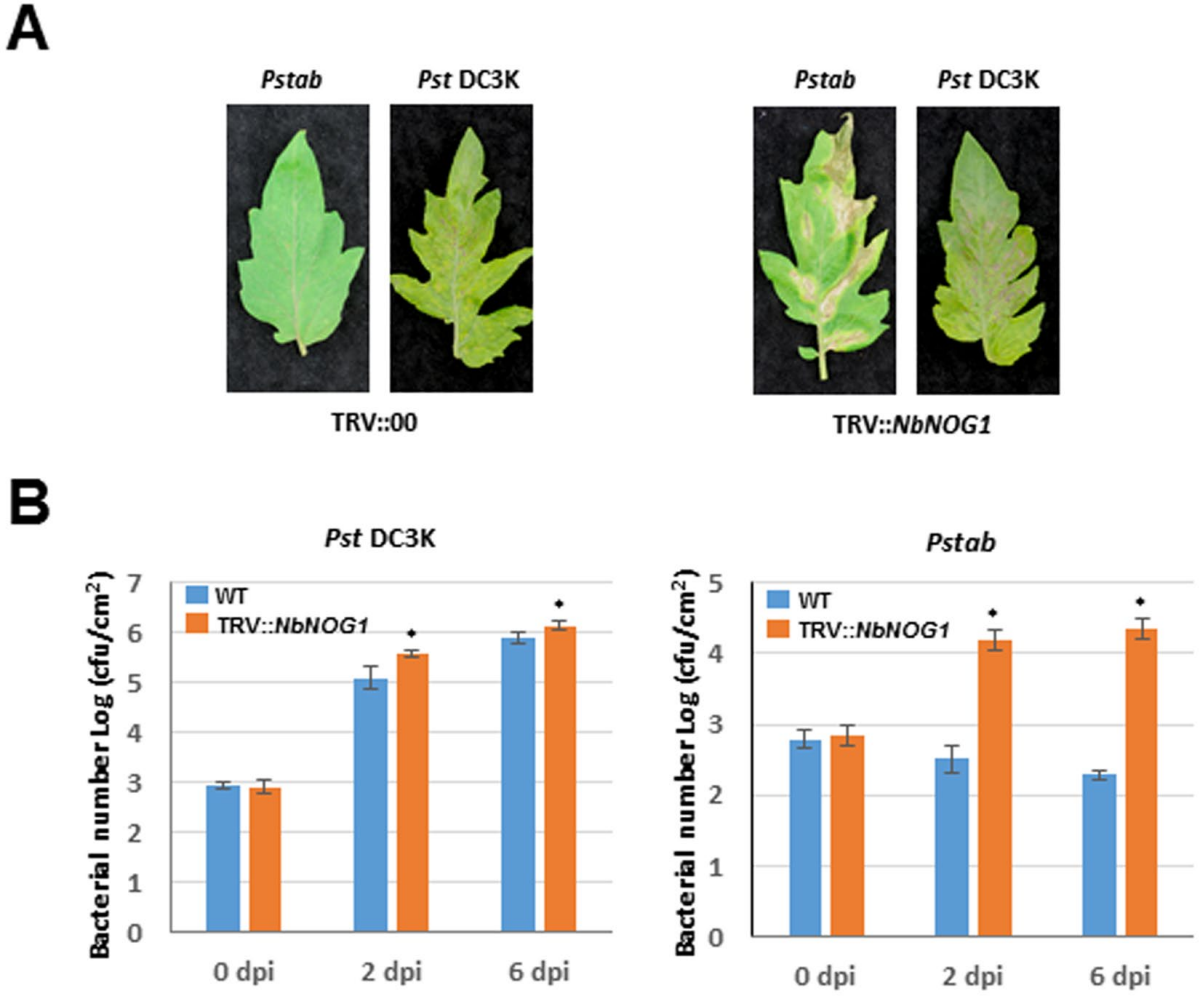
Tomato *SlNOG1–*silenced plants are compromised in nonhost resistance. (**A**) *SlNOG1*-silenced tomato compromised nonhost resistance. TRV::*NbNOG1* and TRV::00 inoculated tomato plants were sprayed with the nonhost pathogen *P*. *syringae* pv. tabaci (*Pstab*) and the host pathogen *P*. *syringae* pv. tomato DC3000 (*Pst* DC3K). Pictures were taken after 5 days after inoculation. (**B**) The bacterial growth of both pathogens was significantly higher in *SlNOG1*-silenced plants than TRV::00 plants. Bacterial growth was measured after 2 and 6 dpi. Bars represent means and standard deviation for three independent experiments. Asterisks represent statistically significant difference between treatments for equivalent time points using Student’s *t*- test (*P* < 0.05).

To determine if *NOG1* is involved in nonhost resistance in other plant species, we used *N. benthamiana NOG1* to silence its orthologous gene in tomato (*SlNOG1*) by VIGS. *SlNOG1*-silenced tomato plants and non-silenced control were inoculated with the tomato nonhost pathogen *P. syringae* pv. tabaci that causes fire blight disease in tobacco. Similar to the findings in *N. benthamiana*, downregulation of *SlNOG1* compromised nonhost disease resistance in tomato resulting in disease symptoms and increased bacterial multiplication when compared to the control (Fig. 2A). Inoculation with the host pathogen *P. syringae* pv. tomato DC3000 caused slightly more severe disease symptoms accompanied with a higher bacterial titer in the *SlNOG1-*silenced plants than in control plants (Fig. 2B). Taken together, these results suggest that *NOG1* is required for nonhost resistance against bacterial pathogens in *N. benthamiana* and tomato.

### *NOG1-1* and *NOG1-2* are members of the small GTP-binding family proteins OBG in Arabidopsis

NbNOG1 showed a high degree of similarity to proteins belonging to the small GTP-binding family protein OBG such as yeast Nog1p (42.7%) and human GTP binding protein 4 (GTPBP4; 48.6%) (Fig. S2A and Table S1). Sequence homologs of NOG1 were identified in a wide range of plant species. Two copies of NbNOG1 or SlNOG1 with nucleotide identity of 99.1% and 97.5% were identified in *N. benthamiana* and tomato, respectively. We identified two Arabidopsis genes, *At1g50920* (*NOG1-1*) and *At1g10300* (*NOG1-2*), as *NbNOG1* homologs. Both genes are 79% identical at the nucleotide level and 76% similar at the amino acid level, suggesting the selection for functional divergence and adaptation. Using the GTPase domain sequence of *NOG1-1* and *NOG1-2*, a total of 10 orthologs were identified in Arabidopsis. Phylogenetic analysis revealed that NOG1-1 and NOG1-2 are highly similar to small GTP-binding family proteins Obg, DRG, and ERG in Arabidopsis (Fig. S2B). Annotation of the *NOG1-2* sequence in The Arabidopsis Information Resource (TAIR; www.arabidopsis.org) showed a 2,064 bps containing two exons and one intron that is predicted to encode a protein of 687 amino acids. However, results from reverse transcription-PCR (RT-PCR) of full length *NOG1-2* followed by c-DNA synthesis and Sanger sequencing showed that no intron sequence was present, and it encodes a truncated protein with 346 amino acids. This was further confirmed by western blot analysis (Figs S3A and S6C). NOG1-2 protein of ~40 kDa was detected by GTPBP4 antibody (N-terminal region) in Arabidopsis and, as expected, His-tag fused NOG1-2 was ~50 kDa. The reason why TAIR annotation shows the presence of an intron in *NOG1-2* is due to the presence of a stop codon at the predicted intron. To investigate if the early termination occurs only in Col-0 or other Arabidopsis ecotypes, NOG1-2 amino acid sequences were examined in 19 representative different ecotypes. Interestingly, the truncated version of NOG1-2 is only present in Col-0, Ler-0, Rsch-4 and Wil-2 (Fig. S3B; Table S2). This early translational termination does not affect the GTPase domain. Furthermore, sequence alignment with NOG1-2 homologs of other eukaryotes suggested that the NOG1-2 start codon begins 87 bps downstream of the start codon annotated by TAIR (Table S1). According to the protein expression results, the 87-bp deletion does not affect translation (Fig. S3A). This 87-bp deleted *NOG1-2* was used for all experiments in this study. In contrast to NOG1-2, NOG1-1 sequences were highly similar among different ecotypes of Arabidopsis.

### Expression of *NOG1-1* and *NOG1-2* are induced by biotic and abiotic stresses

**Figure 3:**
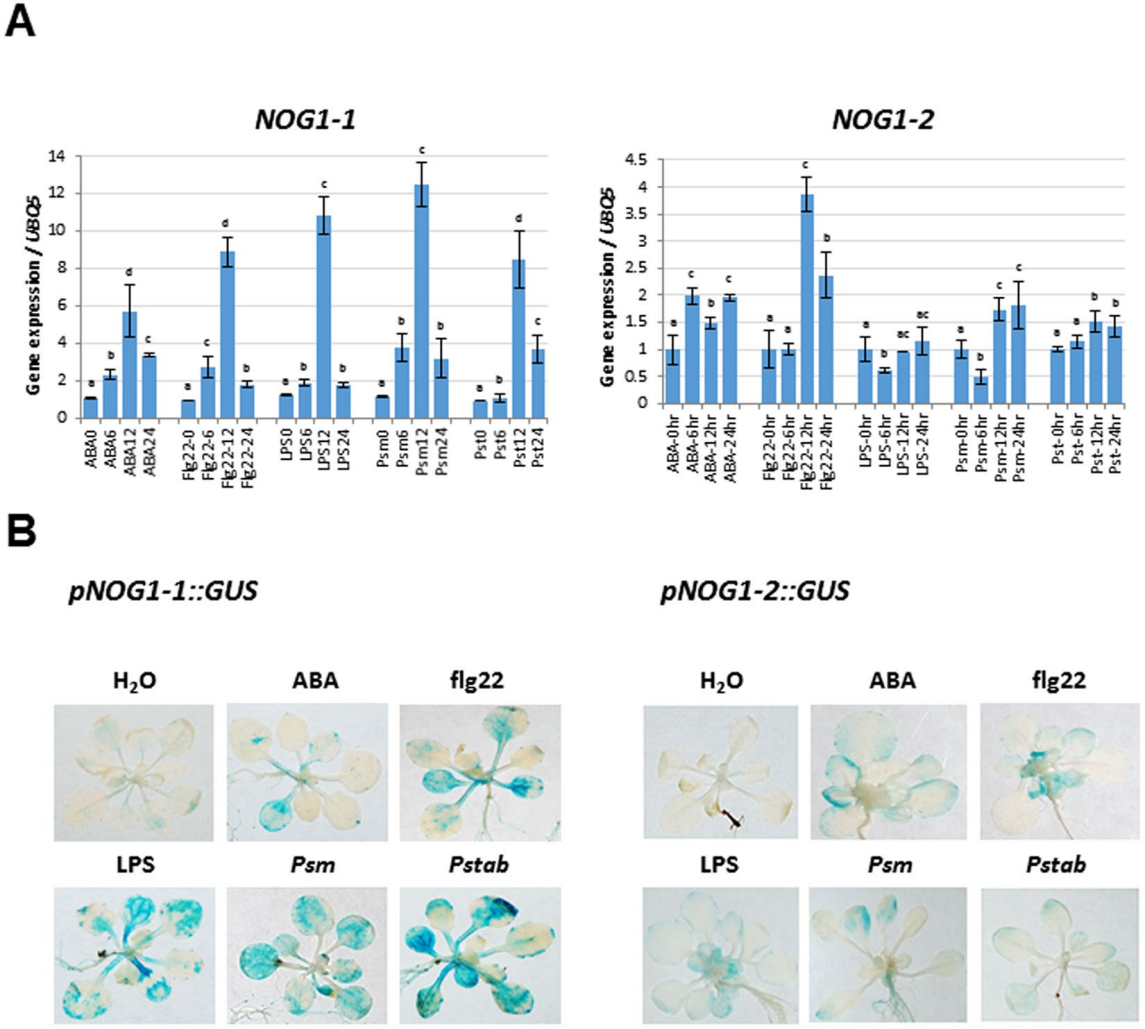
*NOG1-1* and *NOG1-2* are induced by ABA, PAMPs, host and nonhost bacterial pathogens. (**A**) Arabidopsis wild-type (Col-0) plants were individually syringe-infiltrated with ABA (10 µM), Flg22 (20 µM), or LPS (100ng), or flood-inoculated with the pathogens *P. syringae* pv. maculicola (*Psm*) and *P. syringae* pv. tabaci (*Pstab*) at 1 × 10^4^ CFU/ml. RNA was isolated from tissue samples harvested at 0 hr, 6 hr, 12 hr and 24 hr, and qRT-PCR was performed. Bars indicate relative gene expression in comparison with the housekeeping gene *Ubiquitin* (*UBQ5*) and in relation to 0 hr time that was considered as 1. Different letters above bars indicate a statistically significant difference within a treatment using two-way ANOVA (*P* < 0.01). Error bars represent the standard deviation of three biological replicates (three technical replicates for each biological replicate). (**B**) β-Glucuronidase (GUS) staining of *pNOG1-1::GUS* and *pNOG1-2::GUS* in response to ABA, PAMPs, bacterial pathogens. *pNOG1-1::GUS* (left panel) and *pNOG1-2::GUS* (right panel) expression was measured 12 hr after treatment with ABA, flg22, LPS, *Psm* and *Pstab*. Seedlings were flood-inoculated with both pathogens (1.4 × 10^6^ CFU/ml), ABA (10 µM) and PMAPs (flg22: 20 µM, and LPS: 100ng). After 2 hr of GUS straining, plants were washed with sterile water and images were obtained.

The gene expression patterns of *NOG1-1* and *NOG1-2* were determined by quantitative RT-PCR (qRT-PCR) after treating wild-type Col-0 plants with ABA, PAMPs (Flg22 and LPS), host *P*. *syringae* pv. maculicola (*Psm*) and nonhost *P*. *syringae* pv. tabaci (*Pstab*) bacterial pathogens. *NOG1-2* was induced ~4 fold at 12 h post treatment (hpt) with Flg22, ~2 fold with ABA treatment at 6 hpt and ~1.5-fold after treatment with either the host or nonhost pathogens tested (Fig. 3A). *NOG1-1* exhibited a more dynamic expression range and was highly induced at 12 hpt with ABA, Flg22, host and/or nonhost pathogens. Interestingly, at 24 hpt, the induction of *NOG1-1* was reduced by more than 50% (Fig. 3A) in response to each of the treatments.

Arabidopsis lines expressing the β-glucuronidase (*GUS*) reporter gene under the control of *NOG1-1* or *NOG1-2* promoters showed expression of *GUS* in guard cells and hydathodes, which are the natural openings for the entry of bacterial pathogens (Fig. S4A,B). In addition, these lines showed distinct patterns of *GUS* expression of *pNOG1-1-GUS* and *pNOG1-2-GUS* in different tissues (Fig. S4). For example, *NOG1-1* was expressed in the most parts of a flower, while *NOG1-2* expression was only found in flower petal.

To verify the expression of *NOG1-1* and *NOG1-2 in vivo*, the changes in GUS activity in the transgenic plants were determined following treatment of biotic and abiotic stimuli. As shown in Fig. 3A, both *NOG1-1* and *NOG1-2* expression were induced in response to ABA, PAMPs and bacterial pathogens (Fig. 3B). These results suggest that *NOG1-1* and *NOG1-2* are involved in defense responses to both biotic and abiotic stresses.

### *NOG1-1* is necessary for defense responses against bacterial pathogens

As described in Fig. 1, *NbNOG1*- and *SlNOG1*-silenced *N. benthamiana* and tomato plants, respectively, compromised nonhost resistance. The function of *NOG1-1* and *NOG1-2* for nonhost resistance was tested in Arabidopsis. Because *nog1-1* T-DNA insertion mutants were not available, we generated RNA interference (RNAi) lines to downregulate *NOG1-1* expression. Among 23 transgenic lines, two RNAi lines, RNAi2 and RNAi10, that showed ~50% downregulation of *NOG1-1* were selected for further experiments (Fig. S5A). The expression of *NOG1-2* was not altered in *NOG1-1*-RNAi plants (Fig. S6B). Similar to *NbNOG1*- and *SlNOG1*-silenced plants that showed stunted growth, *NOG1-1*-RNAi plants were slightly smaller than wild type (Fig. S5B).

In contrast to *NOG1-1*, a T-DNA insertion line for *NOG1-2*, SALK_043706, was identified and obtained from the Arabidopsis Biological Resource Center. T-DNA insertion is located at the 3′UTR of the *NOG1-2* gene, which presumably disrupts the poly adenylation signal and affects transcript stability (Fig. S6A). The qRT-PCR and western blot experiments showed that *NOG1-2* transcripts and NOG1-2 protein were significantly reduced in SALK_043706 line (Fig. S6B,C). SALK_043706 (*nog1-2*) was transformed with a construct containing the *NOG1-2* native promoter and coding region but without 3′UTR for a complementation experiment. *NOG1-2* expression was slightly increased in the complemented line (*NOG1-2*-comp) but still comparable to the expression of *NOG1-2* in Col-0 (Fig. S6B). In contrast to *NOG1-1*-RNAi plants, *nog1-2* showed a wild-type phenotype. The number of stomata/leaf area was not different in *NOG1-1* RNAi or *nog1-2* plants when compared to Col-0. We generated a double-gene knockdown plant by transforming *nog1-2* with an *NOG1-1*-RNAi construct. Two lines, *nog1-2 NOG1-1*-RNAiA and *nog1-2 NOG1-1*-RNAiB, which showed ~50% *NOG1-1* downregulation, were selected for further experiments (Fig. S5C). In addition, *NOG1-1* was overexpressed in Arabidopsis Col-0 (*NOG1-1* OE).

**Figure 4:**
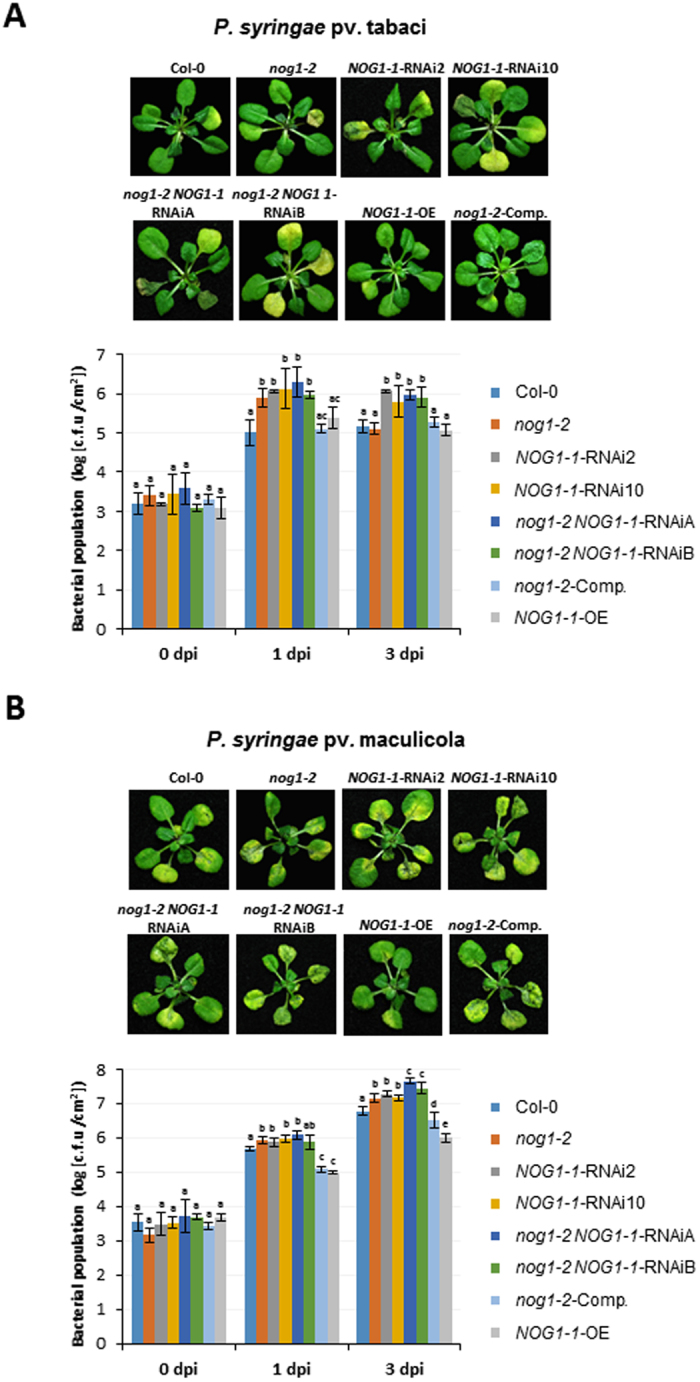
Arabidopsis *NOG1-1-*RNAi but not *nog1-2* plants are compromised in nonhost resistance. (**A**,**B**) Arabidopsis wild type (Col-0), *nog1-2* knockdown line, *NOG1-1-*RNAi, *nog1-2 NOG1-1-*RNAi double-gene knockdown lines, overexpression (*NOG1-1-OE*) and complementation lines (*NOG1-2*-comp) were flood-inoculated with *Pstab* (1.4 × 10^6^ CFU/ml) (A) or *Psm* (1 × 10^4^ CFU/ml) (**B**) to assess disease symptoms (upper panel) and bacterial growth (lower panel) at 1 and 3 days post inoculation (dpi). Different letters above bars indicate a statistically significant difference within a time point using two-way ANOVA (*P* < 0.01). Error bars represent the standard deviation of three biological replications (three technical replicates for each biological replication). All experiments were conducted using T2 lines.

The double-gene knockdown lines along with Col-0, single-gene knockdown and overexpressor lines were flood-inoculated^27^ with *Pstab* (Fig. 4A) or *Psm* (Fig. 4B). *NOG1-1*-*RNAi* lines and the double-gene knockdown lines had ~10-fold increased bacterial growth when compared to Col-0 (Fig. 4A). The *nog1-2* line did not support more growth of *Pstab* at 3 dpi even though ~10-fold increase in bacterial growth was observed at 1 dpi when compared to Col-0 (Fig. 4A). Both *nog1-2* and *NOG1-1*-*RNAi* lines showed slightly enhanced susceptibility to the host pathogen *Psm* by supporting higher bacterial growth (Fig. 4B). Double-gene knockdown lines showed an additive effect in comparison with single-gene knockdown lines for hyper-susceptibility to host pathogen inoculation. Strikingly, *NOG1-1-*OE lines exhibited fewer disease symptoms and harbored fewer bacteria compared to Col-0 (Fig. 4B).

### *NOG1-2* is involved in the regulation of stomatal closure in response to pathogens and abiotic stimuli

**Figure 5:**
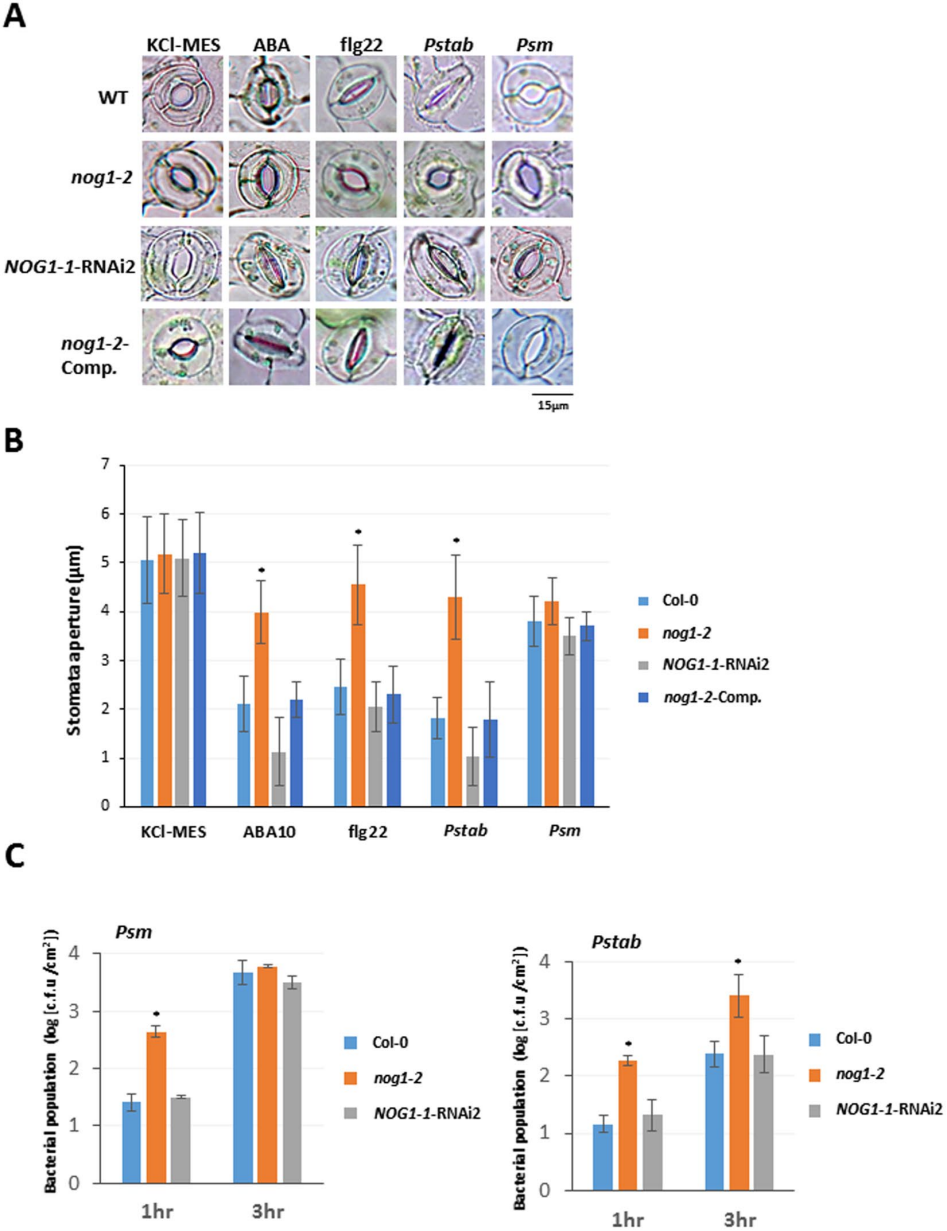
ABA, PAMPs, and nonhost bacterial pathogens induce stomatal closure in *NOG1-2*-dependent manner. (**A**,**B**) The *nog1-2* line impairs ABA-, PAMPs- and nonhost-bacterial-pathogen-induced stomatal closure. To observe stomatal behavior, epidermal peels of Col-0, *nog1-2*, *NOG1-1-RNAi2*, and *NOG1-2* complemented lines were treated with stomata-opening buffer (KCl-MES), ABA (10 µM or 50 µM), flg22 (20 µM), *Pstab* and *Psm* at 1 × 10^4^ CFU/ml. Microscopic images were taken 3 hr after inoculation. The aperture size of stomata was measured after 30 min for ABA, 1 hr for flg22 and LPS, and 3 hr for *Pstab* and *Psm*. Asterisks indicate significant difference by Student’s *t*-test (*P* < 0.05). Error bars indicate standard error for counting 50 stomata/each epidermal peel. Three samples were examined for each treatment, and the experiment was repeated at least three times with similar results. (**C**) Bacterial entry through stomata in *nog1-2* and *NOG1-1-RNAi2* lines. To quantify bacterial entry, detached Arabidopsis leaves from wild-type Col-0 and *nog1-2* and *NOG1-1-RNAi2* were floated in bacterial suspensions (1 × 10^4^ CFU/ml) of the nonhost pathogen (*Pstab*) or host pathogen (*Psm*). After 1 hpi and 3 hpi, leaves were surface-sterilized with 10% bleach, ground, serially diluted and plated on KB media (**B**). After 2 days, the number of bacterial colony was counted. This experiment was repeated three times and showed similar results: five replications in each experiment. Asterisks indicate significant difference by Student’s *t*-test (*P* < 0.05).

*NOG1-1* and *NOG1-2* were induced by ABA (Fig. 3) and therefore the role of these genes in stomatal defense was studied. Arabidopsis epidermal peels were prepared from wild-type Col-0, *nog1-2*, *NOG1-1-RNAi2*, and *NOG1-2*-comp plants and treated with either ABA, Flg22, nonhost (*Pstab*), or host pathogen (*Psm*). In response to ABA, Flg22, and *Pstab*, *NOG1-1-RNAi2*, and *NOG1-2*-comp lines closed stomata similar to Col-0, while the *nog1-2* stomata were not completely closed (Fig. 5A). Treatment with host pathogen, *Psm*, caused stomata to remain open in all the lines tested, because this pathogen is known to produce COR that can reopen stomata. Quantification of these results was obtained by measuring the stomatal aperture (Fig. 5B). The aperture size of stomata in Col-0, *NOG1-1-RNAi2*, and *NOG1-2*-comp lines decreased by 50% to 80% upon treatments that close stomata, while stomatal aperture in *nog1-2*, remarkably, was only reduced by 10% to 30% (Fig. 5B).

The observation that *nog1-2* is defective in closing stomata during biotic stress suggested that *nog1-2* could enable more pathogen entry. To test this hypothesis, epidermal peels of *nog1-2*, *NOG1-1-RNAi2*, and Col-0 were individually incubated with *Psm* and *Pstab* expressing *GFPuv* ^28^, respectively. The bacterial entry in *nog1-2* and Col-0 plants was quantified at 1 hour post inoculation (hpi) and 3 hpi. The number of host bacterial cells (*Psm*) was greater in *nog1-2* at 1 hpi but was not different than wild-type and *NOG1-1-RNAi2* at 3 hpi since the host pathogen was able to reopen stomata (Fig. 5C). Number of nonhost bacterial cells (*Pstab*) inside *nog1-2* leaves was ~10-fold higher than in Col-0 and *NOG1-1*-RNAi line at both 1 and 3 hpi (Fig. 5C). In contrast to *nog1-2*, *NOG1-1*-*RNAi* lines did not show any difference in entry of bacteria through stomata when compared to Col-0 (Fig. 5C). This agrees with the results shown in Fig. 5A that the stomata closure in *NOG1-1-RNAi2* in response to ABA, flg22, and nonhost bacterial pathogen (*Pstab*) was not altered even though *NOG1-1* was highly expressed in guard cell (Fig. S4). It is possible that *NOG1-1* may have a role in stomatal aperture regulation and/or development, but the transcript reduction levels in the RNAi lines is not sufficient to observe defects in stomatal aperture regulation.

### *NOG1-2* has GTPase activity and positively regulates bacterial pathogen- and abiotic-mediated guard cell signalling

**Figure 6:**
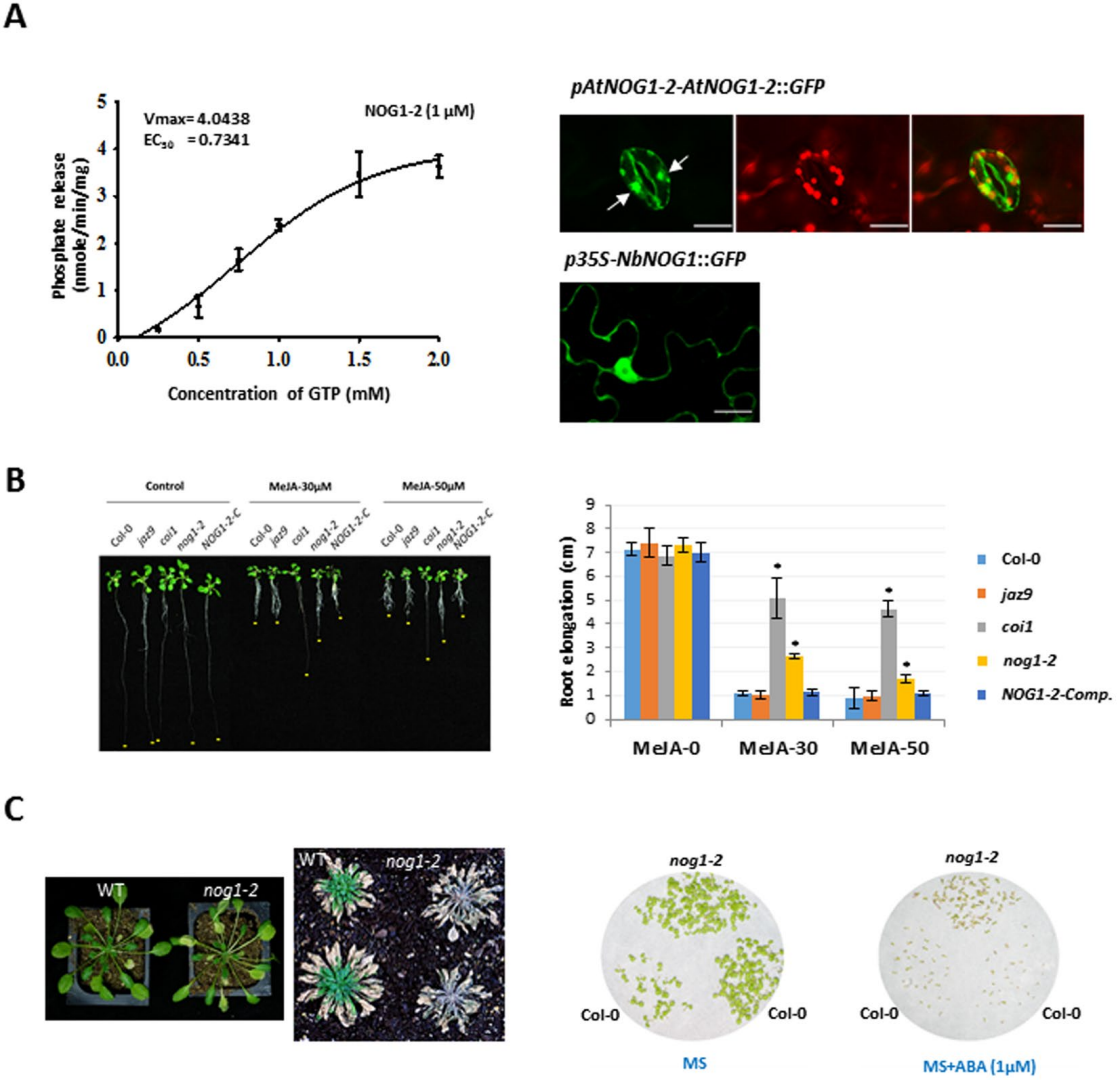
NOG1-2 has GTPase activity and is functionally involved in JA- and ABA-mediated signaling pathway. (**A**) Left Panel: Rate of Pi release due to the GTPase activity of NOG1-2 protein (1 µM) in the presence of varying concentrations of GTP. Experiments were repeated three times, and data were averaged. Error bars represent the mean ± S.E. Right panel: The transgenic Arabidopsis or *N. benthamiana* plants expressing *AtNOG1-2-GFP* under native promoter of *AtNOG1-2* or *NbNOG1*-*GFP* under *35 S promoter*, respectively. Arrows represent nuclei in guard cells. One week of seedlings were observed for the localization of AtNOG1-2 under confocal laser microscopy. Scale bar is 10 µM. *Atnog1-2* is less sensitive to JA than Col-0. (**B**) *Atnog1-2* line, compared to wild-type Col-0, is less sensitive to JA. Seeds of different Arabidopsis lines were grown in ½ MS medium plates with or without 30 and 50 µM of MeJA, and 7 days later root lengths were measured. Three independent experiments were done, with at least 10 seedlings for each line. Bars represent means ± SD. Asterisks indicate significant difference from Col-0 by Student’s *t*-test (*P* < 0.05). (**C**) The mutation of AtNOG1-2 increases sensitivity to drought stress and ABA. Wild-type (Col-0) and *nog1-2* plants were grown for four weeks (21 °C/14 hr day and 18 °C/10 hr night), then plants were dehydrated until drought symptom appeared. After leaves were completely collapsed, plants were re-watered to revive. *nog1-2* seedlings are less sensitive to ABA. Seedlings of Col-0 and *nog1-2* were grown in MS or MS with ABA (1uM) for 2-weeks.

To examine the role of NOG1-2, the biochemical activity of recombinant and purified AtNOG1-2 was assessed in a hydrolysis and phosphate release assay (Fig. 6A, left panel and Fig. S6D). The JAZ9 protein that has been shown to play a role in stomatal closure^29^, which has no known GTPase domain, was used as negative control. Our results show that NOG1-2 has GTP-binding and GTPase activity. Furthermore, *NOG1-2* was strongly expressed in guard cells of the Arabidopsis transgenic plants expressing *AtNOG1-2-GFP* fusion driven by *AtNOG1-2* promoter (Fig. 6A, right panel). NOG1-2 was localized to the nucleus in guard cells of Arabidopsis. In *N. benthamiana*, NbNOG1-GFP (*35 S::NOG1*) was localized to the nuclei and cytoplasmic membrane (Fig. 6A).

To further examine the involvement of NOG1-2 in JA- and ABA-mediated signaling pathway, the sensitivity of *nog1-2* to MeJA and ABA was tested. As reported earlier^30^, several JAZ (*jaz9* used in this study) mutants showed sensitivity to MeJA because of the functional compensation by other JAZs, while *coi1* mutant showed less sensitivity to MeJA (Fig. 6B). Interestingly, *nog1-2* also showed reduced sensitivity to MeJA. It was also found that *nog1-2* plants are more susceptible to drought stress and less response to ABA, suggesting that NOG1-2 is involved in JA and ABA signaling pathway (Fig. 6C).

In order to dissect whether *NOG1-2* is closely related to other genes involved in guard cell signaling, the gene expression profiling was conducted in *nog1-2* lines in response to ABA, coronatine (COR), and host and nonhost bacterial pathogens (Fig. S7). A total of 12 functionally characterized genes for guard cell signaling such as *OST1*, *OST2*, *rbohD*, *MPK4*, *MPK9*, *MPK12*, *ABI1*, *SLAC1*, *RIN4*, *SLAH3*, *CPK4* and *CPK6* were determined for the expression patterns upon exposure to both abiotic and biotic stimuli in *nog1-2* lines. After ABA treatment, *OST2* expression was significantly increased in Col-0 at both 12 and 24 hr, but the expression was decreased in *nog1-2* at 24 hr. The expression of *MPK4*, *MPK9*, *ABI1*, and *CPK6* was highly upregulated in Col-0 at 12 hr, while these genes were not notably induced in *nog1-2*. After treatment of COR, *rbohD*, *MPK4*, *MPK12*, and *SLAC1* were rapidly induced in Col-0 at 12 hr, but not found in *nog1-2*. *MPK9* and *RIN4* expression was notably decreased in *nog1-2* at 24 hr. After host pathogen *(Psm*) infection, the expression of MPK9 was significantly increases in Col-0 at 24 hr; however, this gene was not upregulated in *nog1-2*. The similar pattern was also found for *MPK12*.

### Transcriptome analysis reveals the regulation of *NOG1-1* and *NOG1-2* in plant innate immunity against bacterial pathogens

The transcriptome analysis was performed in Col-0, *NOG1-1* RNAi, and *nog1-2* lines without any treatment of biotic and abiotic stimuli using Affymetrix GeneChip® Arabidopsis Genome Arrary (Affymetrix). A total of 161 genes were identified as differentially expressed genes (DEGs) in *NOG1-1* RNAi and *nog1-2* lines compared to Col-0, respectively (Table S3). For *nog1-2*, only 14 DEGs were identified, nine for upregulation and five for downregulation. All genes are highly related in the signaling pathway of biotic and abiotic stress responses.

**Figure 7:**
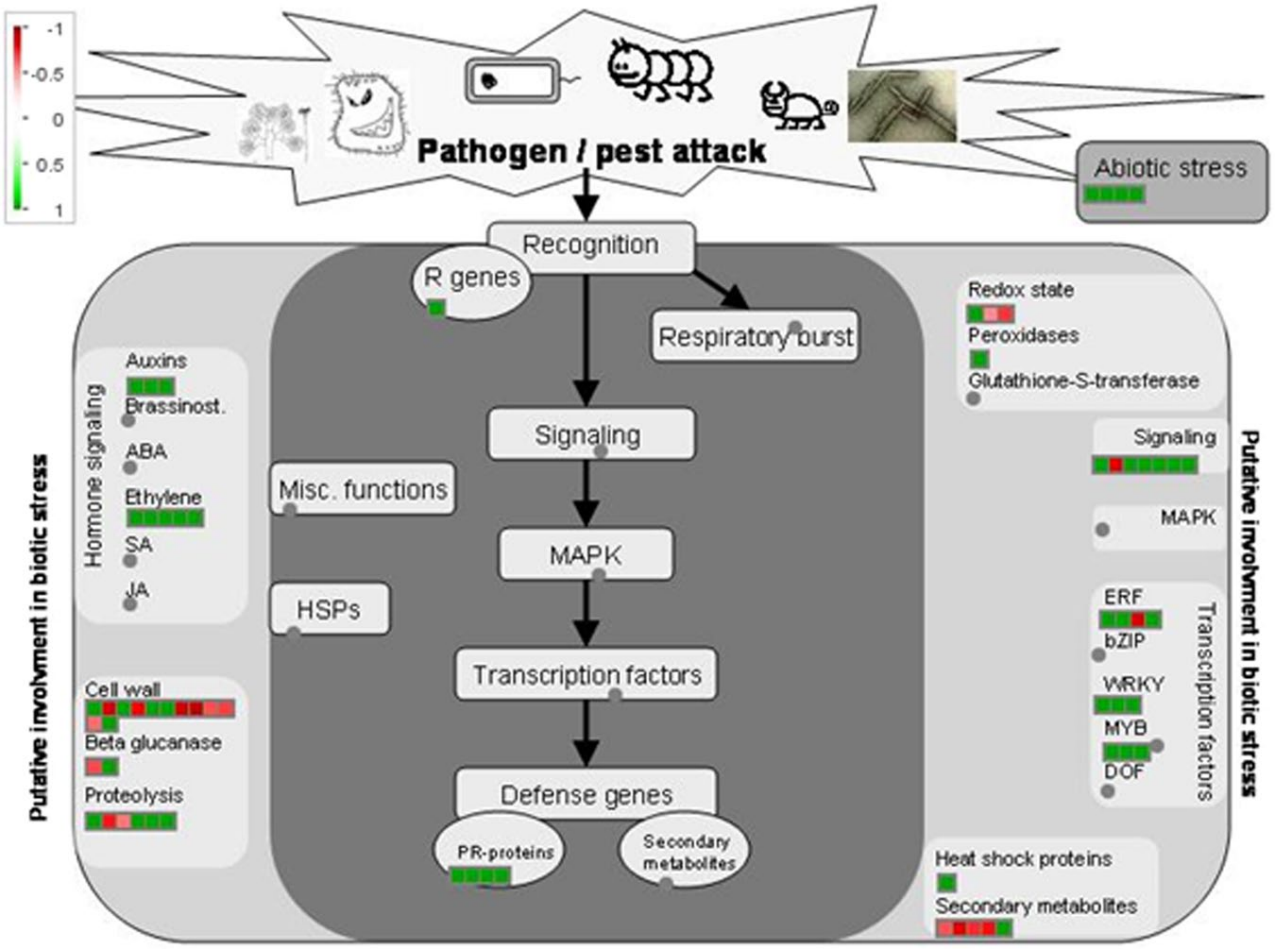
MAPMAN visualization of Arabidopsis Affymetrix data describing the differentially expressed genes involved in plant defense responses in NOG1-1 RNAi and *nog1-2* lines. The Affymetrix microarray analysis showed a number of up- and down-regulated genes in *NOG1-1* RNAi and *nog1-2* lines compared to wild-type (Col-0) without treatment. MAPMAN was used to analyze the gene function and biological pathways of *NOG1-1* and *NOG1-2*. Four-week old seedlings grown on half MS media were collected for RNA extraction. Three biological replicates were used for each *NOG1-1* RNAi and *nog1-2* without any treatments. Color patterns from red (upregulation) to green (downregulation) indicate the change of gene expression.

MAPMAN software was used to visualize the DEGs of *NOG1-1* RNAi and *nog1-2* to determine their putative role in plant defense. Because of very low number of DEGs in *nog1-2*, DEGs for both *NOG1-1* RNAi and *nog1-2* lines were pooled for the analysis. The DEGs represented in the Arabidopsis microarray were classified into different functional groups using automated and manual annotation. The MAPMAN analysis identified that the common DEGs in *NOG1-1* RNAi and *nog1-2* were highly responsive to biotic and abiotic stresses (Fig. 7). Most of down-regulated genes in both *NOG1-1 RNAi* and *nog1-2* are involved in the signaling pathways for abiotic and biotic defense responses. The number of DEGs was significantly higher in *NOG1-1* RNAi than in *nog1-2*.

## Discussion

This study identified a small GTP-binding protein (GTPase), *NOG1*, as a novel player in plant immunity against bacterial pathogens. Two copies of this gene, *NOG1-1* and *NOG1-2*, exist in plants, and are required for nonhost resistance associated with apoplastic and stomatal defense. Stomatal closure in plants can be triggered by bacterial pathogens and PAMPs such as flg22 and LPS^4, 5, 7^. The guard cell signaling pathway involved in PAMPs- or pathogen-induced stomatal closure is still not fully understood. Only a few proteins, such as FLS2, COI1, MYC2 and MPK4, have been studied with respect to stomatal closure in response to phytobacterial pathogens^31^. Also, Penetration 3 (PEN3) has been demonstrated for stomatal defense against fungal pathogens in Arabidopsis^32, 33^. The results reported here suggest that NOG1-2 may be an additional key regulator of stomatal closure in response to biotic and abiotic stimuli. Interestingly, NOG1-1 doesn’t seem to play a major role in regulating stomatal closure but is involved in apoplastic defense against bacterial pathogens, indicating a possible interplay between NOG1-1 and NOG1-2 in plant innate immunity, such as regulation of stomatal opening and induction plant defense responses.

Small GTPases have been studied extensively for their role in cellular development and regulation of signal transduction in plants^13^. More than 100 small GTPases are known from higher eukaryotes, which are generally classified into Ras, Rho, Rab, Sar1/Arf and Ran families^34^. Rho and Rab small GTPases have been widely studied for their role in defense signaling against fungal and bacterial pathogens^35^. *NOG1-1* and *NOG1-2* encode small GTPases that belong to OBG family whose function in plants has never been investigated. In mammals and yeast, the orthologs of *NOG1* are *GTPBP4* and *Nog1p*, and essential for ribosome biogenesis and cell viability^23^. Both GTPBP4 and Nog1p are known to be localized to the nucleus^24^. GTPBP4 orthologs are highly conserved within their GTPase domains (Fig. S2A) and found in many eukaryotes (http://www.genecards.org). Interestingly, *NOG1/GTPBP4* orthologs are always present as a single copy in mammals, insects, and yeast, while two homologs are found in monocot and dicot plant species (http://www.phytozome.net). Only one copy of the *NOG1* ortholog is present in two algae species (*Chlamydomonas reinhardtii*; XM_001698344 and *Guillardia theta*; XM_001698344), but two homologs are present in moss (*Physcomitrella patens* subsp. *Patens*; XM_001698344 and XM_001761522). This finding suggests that higher plant species may need an additional copy of *NOG1* for a plant-specific function, such as regulation of stomatal opening and early defense responses specific to plants.

The Arabidopsis genome has genes for 12 JAZ family proteins. It has been reported that single gene mutations in genes encoding JAZ2, JAZ5, JAZ7 or JAZ9 did not result in JA insensitivity as in *coi1* mutants, suggesting functional redundancy among JAZ proteins in Arabidopsis^30^. Furthermore, Arabidopsis *jaz1 jaz2* double mutant did not alter JA signaling^36^. In Arabidopsis, several JAZs have been shown to interact with COI1^37^, and represses the MYC2 transcription factor to regulate JA-mediated stomatal closure^10^. COI1 functions as a receptor for JA and recruits JAZ proteins for ubiquitination and degradation via the 26S proteosome. It is uncertain if NOG1-2 function for stomatal closure is associated with JAZ/COI1-mediated JA signaling pathway. MYC2 is another key component of the JA signaling pathway^38^. It has been reported that MYC2 interacts with all 12 JAZ proteins, further suggesting the redundant function of JAZs^39^. MYC2 induces JA-responsive genes, and its activity is reduced by JAZ proteins. MYC2 has been shown to be phosphorylated by MPK6 in the regulation of seedling development and photomorphogenesis^40^. Figure 7 shows that several genes involved in MAPK signaling pathway are differentially expressed in *NOG1-1* RNAi and *nog1-2* lines. It will be interesting to determine if NOG1-2 can be phosphorylated by a kinase. There is evidence for the phosphorylation of small GTPases by kinases that enhance GTPase activity^41^.

As shown in Fig. 6, *nog1-2* plants are more susceptible to drought stress and less sensitive to ABA, indicating the involvement of NOG1-2 in the guard cell signaling pathway. The expression of *OST2*, *MPK4*, *MPK9*, *ABI1*, and *CPK6*, which are key players in guard cell ABA signal transduction, was significantly altered in *nog1-2* line after ABA treatment (Fig. S7). This finding suggests that NOG1-2 may be a key element upstream of guard cell regulating and ABA-induced genes and interplay with a complex network of ABA signaling pathways. MPK4 is known to negatively regulate stomata open/closure against bacterial pathogens^42^. Our study also showed the expression changes of MPK4 in response to PAMPs and bacterial pathogens in *nog1-2* line. It has been known that MPK9 and MPK12 are highly expressed during ABA-induced and H_2_O_2_-induced stomatal closure^43^. These two genes are differently expressed in *nog1-2* line when compared to Col-0, suggesting that MPK9 and MPK12 function in bacterial pathogen-induced guard cell signaling pathway.

In conclusion, we identified a novel role of NOG1 in plant innate immunity and it would be important to further investigate the mechanism of plant defense response mediated by NOG1. More interestingly, we identified the novel function of NOG1-2 for stomatal closure in response to biotic and abiotic stimuli. This warrants further investigation for the role of NOG1-2 in stomatal regulation through JA and ABA signaling. Nevertheless, identification of NOG1 as one of the key regulators of stomatal aperture and plant innate immunity will become an important avenue to better understand plant response to biotic and abiotic stresses.

## Methods

### Virus-induced gene silencing in *N*. *benthamiana* and tomato plants

VIGS library used in this study for forward genetics screening was constructed using RNA from various biotic and abiotic stress inducing elicitor treated *N. benthamiana* plants. *Agrobacterium tumefaciens* GV2260 containing TRV1, TRV2::00 and TRV2::*NOG1* was grown overnight on LB medium containing antibiotics (rifampicin, 25; kanamycin, 50) at 28 °C. Bacterial cells were harvested and resuspended in induction medium (10 mM MES pH 5.5; 200 uM acetosyringone), and incubated at room temperature on an orbital shaker for 5 hr. Bacterial cultures containing TRV1 and TRV2 were mixed in equal ratios (OD_600_ = 1) and infiltrated into *N. benthamiana* or tomato leaves using a 1 ml needleless syringe^44^. The infiltrated plants were maintained in a greenhouse and used for studies 15 to 21 days post-infiltration. Table S6 has all the primer information used in this study.

### Hypersensitive response analysis

For nonhost pathogens-dependent HR, the bacterial suspension in MES buffer (MES 10Mm, pH6.5) was syringe-infiltrated to fully expanded *N. benthamiana* leaves for determining nonhost HR cell death. For R/Avr-dependent HR, leaves were infiltrated with a mixture of *Agrobacterium tumefaciens* expressing *Avr* genes and its complementary *Cf* or *Pto* gene using a sterile needleless syringe. *Pto* and *AvrPto*, or *Cf9* and *AvrCf9* constructs were mixed to 1:1 ratio before infiltration to *N. benthamiana* leaves. The agro-inoculated plants were maintained under standard growth condition, and HR cell death in the inoculated area was investigated and photographed.

### Plant growth, pathogen inoculation, and bacterial growth assay

*N. benthamiana* and tomato plants were grown in greenhouse. Silenced and control *N. benthamiana* plants were inoculated with appropriate bacterial pathogens. Bacterial strains were grown at 28 °C on KB medium containing antibiotics in the following concentrations (μg/ml): rifampicin, 50; kanamycin, 25; chloramphenicol, 25 and spectinomycin, 25 for 24 hr. To prepare bacterial inoculum, the culture media were centrifuged at 5000 rpm for 10 min and resuspended in water for bacterial growth assays using vacuum infiltration and spraying. The inoculated plants were then incubated in growth chambers at 90 to 100% relative humidity for the first 24 h.

*Arabidopsis thaliana* T-DNA insertion lines: SALK_043706 and SALK_072852 containing insertions in *NOG1-2* were obtained from http://signal.salk.edu/cgi-bin/tdnaexpress. Wild-type Col-0 and T-DNA insertion lines were grown in 1/2 MS plates in growth chamber at 21 °C with a 14-h photoperiod and a light intensity of about 100 uE m^−2^ sec^−1^. Four-week-old plants were inoculated with appropriate host or nonhost bacterial pathogens, and bacterial growth was measured. For the bacterial growth assays in *N. benthamiana* and tomato, leaf samples from inoculated leaves at specific time points after inoculation were collected by using a 0.5 cm leaf puncher. Leaf tissues were ground in sterile water, serially diluted and plated on KB plates supplemented with appropriate antibiotics. For the bacterial growth assays in Arabidopsis after flood-inoculation, inoculated leaves were surface-sterilized with 15% H_2_O_2_ for 3 min to eliminate epiphytic bacteria and then washed with sterile distilled water. The leaves were then homogenized in sterile distilled water, and serial dilutions were plated onto KB medium containing antibiotics. Bacterial growth was evaluated in three independent experiments.

### Stomatal aperture measurements and bacterial entry assay

The stomatal aperture measurement experiments were performed by following the protocol available at Melotto lab, University of California Davis (http://melotto.ucdavis.edu/protocol_stomatal.htm) and the previous study^7^. Briefly, plants were conditioned to open stomata by placing plants under fluorescence light for at least 3 hr. Epidermal peels were then immediately floated on stomata-opening buffer (10 mM MES-Tris pH 6.1, 10 mM KCl) for 3 hr. At various time points, the epidermal peels were treated with ABA, flg22, LPS and bacterial pathogens. Epidermal peels were observed under Nikon light microscope.

To determine bacterial entry via stomata, detached leaves from 2-week-old seedlings grown in ½ strength MS medium were floated on bacterial suspension. After 1 hr or 3 hr incubation, leaf surfaces were sterilized using 10% bleach (Clorox), then observed under a fluorescent microscope or plated on KB medium after serial dilutions.

### *In vitro* GTPase activity assay and phosphate release assay

The GTPase activity of NOG1-2 was also evaluated using the ENZchek phosphate release assay kit (Thermo Fisher Scientific, NY). Phosphate (Pi) production was detected as a change in absorbance at 360 nm using a Spectramax M2 spectrophotometer (Molecular Devices, Sunnyvale, CA). The amount of Pi released was estimated from the corresponding values obtained with a standard curve. Data was plotted as nanomoles of Pi released min^−1^mg^−1^ and fitted using nonlinear regression in SigmaPlot 11.0.

### Histochemical and fluorescent microscopy analyses

To determine the expression patterns of *NOG1-2* and *NOG1-1*, the promoters of *NOG1-2* (1.2 kb) and *NOG1-1* (0.9 kb) were fused to *GUS* reporter gene. *NOG1-1::GUS* and *NOG1-2::GUS* transgenic seedlings were incubated with GUS staining solution at 37 °C. Staining was discarded and chlorophyll cleared by washing with 70% ethanol and keeping the leaves in ethanol for 72 hrs. GUS activity was analyzed by bright-field transmitted light microscopy, and images were taken by digital camera (Nikon). Confocal analysis of GFP expression was performed using confocal microscope (Biorad, CA).

### Development of transgenic lines

To complement the *nog1-2* knockdown line, the full length of *NOG1-2* coding region was cloned into pMDC162, controlled by *NOG1-2* native promoter. This construct was transformed to GV3101, and transferred into *nog1-2* using Arabidopsis floral dip transformation. To knock-down *NOG1-1* in Col-0, the partial sequence of *NOG1-1* (approximately 400 bp) were selected using pssRNAit program (http://plantgrn.noble.org/pssRNAit/). This fragment was cloned into RNAi vector (Invitrogen, NY) and transformed using Arabidopsis floral dip transformation. To make double-gene knockdown line of *NOG1-2*, *NOG1-1*, *NOG1-1* RNAi construct was transformed into *nog1-2*. To examine the localization of NOG1-2, the full length coding region of both genes were cloned into either pMDC45 or pMDC83.

### RNA extraction and quantitative real-time PCR

Total RNA was purified from Arabidopsis leaves infiltrated with water (mock control), nonhost pathogen *P. syringae* pv. *tabaci* (*Pstab*), or host pathogen *P. syringae* pv. *maculicola* (*Psm*). Total RNA was extracted using TRIzol (Invitrogen), and 2 treated or inoculated leaves were pooled to represent one biological replicate. Total RNA was treated with DNase I (Invitrogen), and 1 μg RNA was used to generate cDNA using Superscript III reverse transcriptase (Invitrogen) and oligo d(T)15–20 primers. The cDNA (1: 20) was then used for real-time quantitative PCR using Power SYBR Green PCR master mix (Applied Biosystems, Foster City, CA, USA) with an ABI Prism 7900 HT sequence detection system (Applied Biosystems). Primers specific for *AtUBQ5* was used to normalize small differences in template amounts. Average Cycle Threshold (CT) values calculated using Sequence Detection Systems (version 2.2.2; Applied Biosystems) from duplicate samples were used to determine the fold expression relative to controls. All primers used are shown in Table S4.

### Transcriptome analysis of *nog1-1* and *nog1-2* using Arabidopsis microarray

Arabidopsis seedlings were grown for seven days on ½ MS in controlled conditions with a 16 hr light, 8 hr dark cycle at 24 °C. Total RNA from three biological replicates of *NOG1-1* RNAi, *nog1-2*, and Col-0 leaves were isolated and cleaned by using the Rnaeasy MinElute Cleanup Kit (Qiagen, WN) and used for two-channel microarray. RNA labelling and hybridization to Affymetrix ATH1 arrays were performed as described in the Affymetrix manual. Data normalization between chips was conducted using RMA (Robust Multichip Average)^45^. Gene selections based on Associative T-test were made using Matlab (MathWorks, Natick, MA)^46^. In this method, the background noise presented between replicates and technical noise during microarray experiments was measured by the residual presented among a group of genes whose residuals are homoscedastic. Genes whose residuals between the compared sample pairs that are significantly higher than the measured background noise level were considered to be differentially expressed. A selection threshold of 2 for up-regulated and 1.5 times for down-regulated and a Bonferroni-corrected P value threshold of 2.19202E-06 were used for further analysis. The Bonferroni-corrected P value threshold was derived from 0.05/N in these analyses, where N is the number of probes sets (22810) on the chip.

### Data Availability Statement

All the data presented in the manuscript will be made publicly available.

## Electronic supplementary material

Supplementary Data

Supplementary Table S1

Supplementary Table S3
