# Supplementary material for: The small GTPase, nucleolar GTP-binding protein 1 (NOG1), has a novel role in plant innate immunity
Source: Sci Rep. 2017 Aug 23;7:9260. doi: 10.1038/s41598-017-08932-9 (PMC5569028; doi:10.1038/s41598-017-08932-9)
Supplement: Supplementary file 1 — Supplementary Data [file 41598_2017_8932_MOESM1_ESM.pdf]

The small GTPase, nucleolar GTP-binding protein 1 (NOG1), has a novel role in plant innate immunity

Seonghee Lee[1,2], Muthappa Senthil-Kumar[1,6], Miyoung Kang[3], Clemencia M. Rojas[1,5], Yuhong Tang[1], Sunhee Oh[1], Swarup Roy Choudhury[4], Hee-Kyung Lee[1], Yasuhiro Ishiga[1,7], Randy D. Allen[3], Sona Pandey[4] and Kirankumar S. Mysore[1*]

[1] Noble Research Institute, LLC., Ardmore, Oklahoma, USA

[2] Gulf Coast Research and Education Center, Institute of Food and Agricultural Science, University of Florida, Wimauma, Florida, USA

[3] Institute for Agricultural Biosciences, Oklahoma State University, Ardmore, Oklahoma, USA

[4] Donald Danforth Plant Science Center, St. Louis, Missouri, USA

[5] Current Address: Department of Plant Pathology, University of Arkansas, Fayetteville, Arkansas, USA

[6] Current Address: National Institute of Plant Genome Research, Aruna Asaf Ali Marg, New Delhi, India

[7] Current Address: Department of Plant Pathology, University of Tsukuba, Ibaraki, Japan

*Correspondence to: Kiran Mysore, Noble Research Institute, 2510 Sam Noble Pky. Ardmore, Oklahoma 73401 U.S.A.

Phone: 580-224-6740 email: ksmysore@noble.org

Figure S1

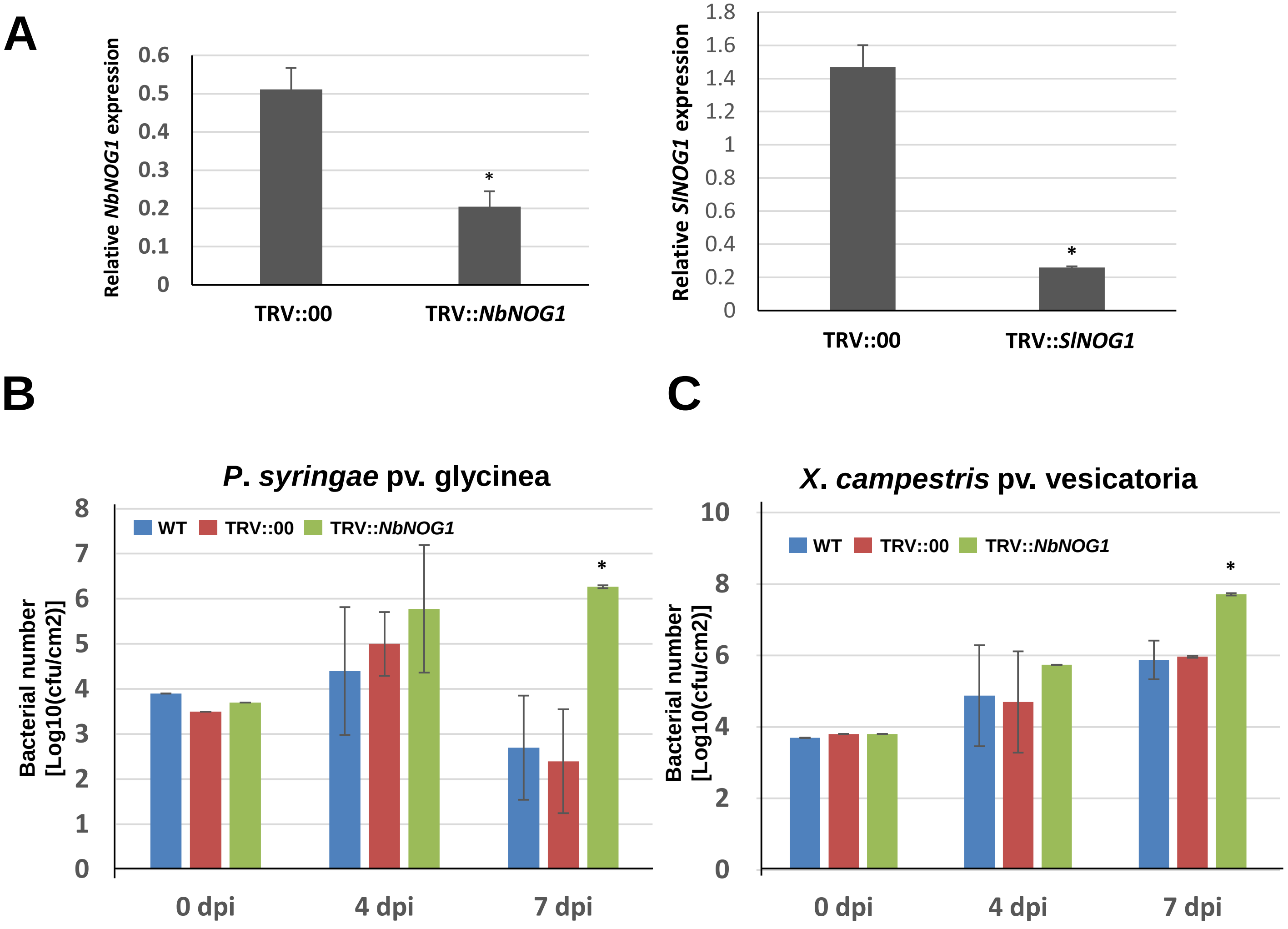

Figure S1. *Nicotiana benthamiana NbNOG1*-silenced plants are compromised in nonhost disease resistance against different nonhost pathogens such as *P. syringae* pv. glycinea and *X. campestris* pv. vescatoria. (A) The level of down-regulation of *NbNOG1* was quantified in control (TRV:00) and *NbNOG1*-silenced *N. benthamiana* plants. The expression of *NbActin* was used as internal control. (B) *NbNOG1*-silenced (TRV::*NbNOG1*) and non-silenced control (TRV::00) *N. benthhamiana* plants were vacuum-infiltrated with *P. syringae* pv. glycinea (B) and *X. campestris* pv. vesicatoria (C). Bacterial growth was monitored at 0, 4 and 7 days post-inoculation.

Figure S2

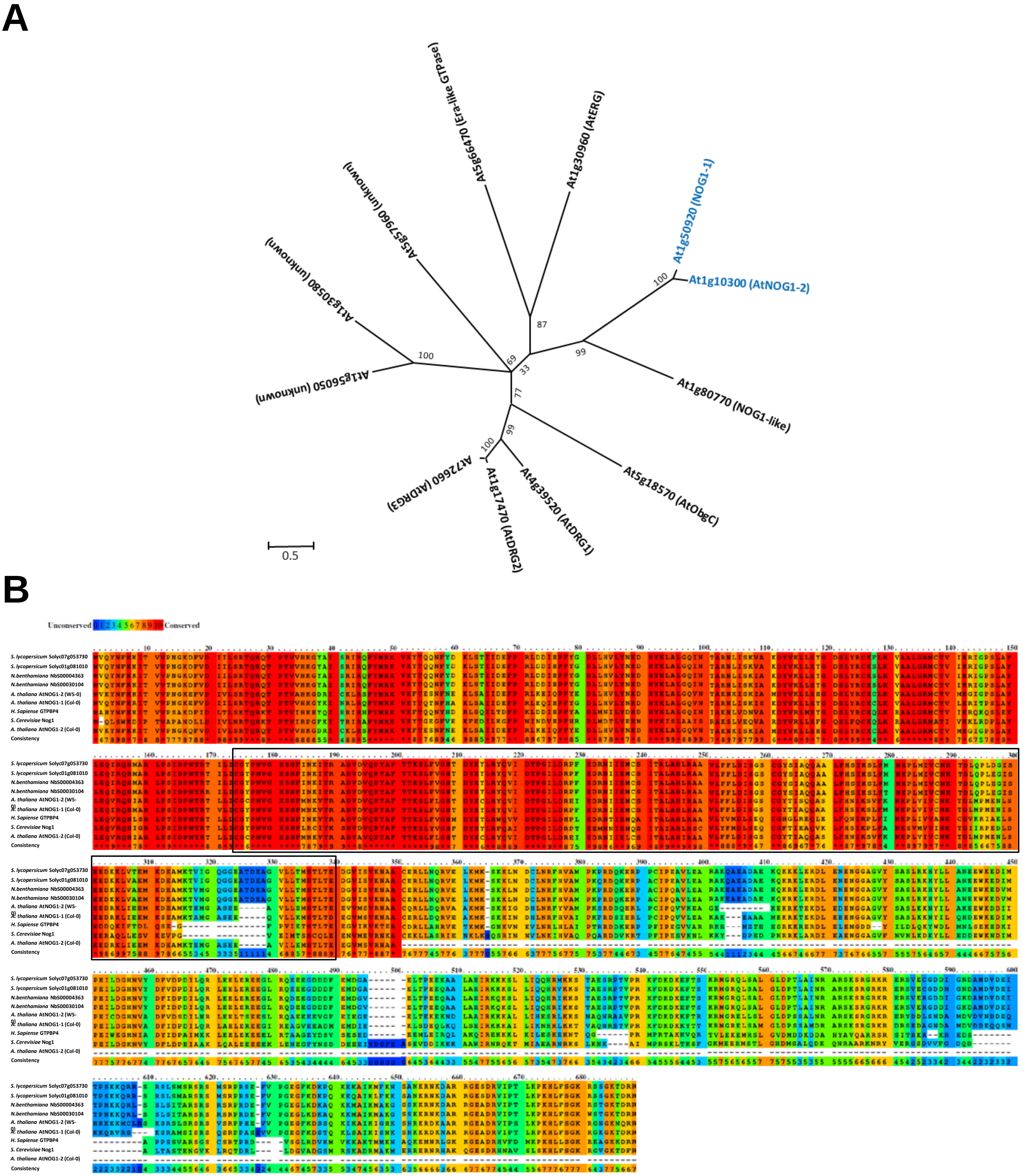

Figure S2. Arabidopsis NOG1-1 and NOG1-2 are small GTP-binding family proteins Obg, DRG, and ERG. (A) Arabidopsis proteins with sequence similarity to NOG1-1 and NOG1-2 were retrieved from the Arabidopsis genome and alignment was generated using ClustalW and the neighbor-joining tree was created. Branch lengths are proportional to the estimated evolutionary distance. Bootstrap values are included. (B) NOG1-1 and NOG1-2 are highly conserved among different organisms and have sequence similarity to the small GTP-binding family proteins OBG Amino acid sequence alignment of NOG1-1 and NOG1-2, and orthologous genes in *N. benthamiana*, tomato, yeast and human. Sequence similarities are represented by different colored boxes. The predicted domain for GTPase is marked by a black box. Sequence alignment was generated using the PRALINE program (http://www.ibi.vu.nl/programs/pralinewww/).

Figure S3

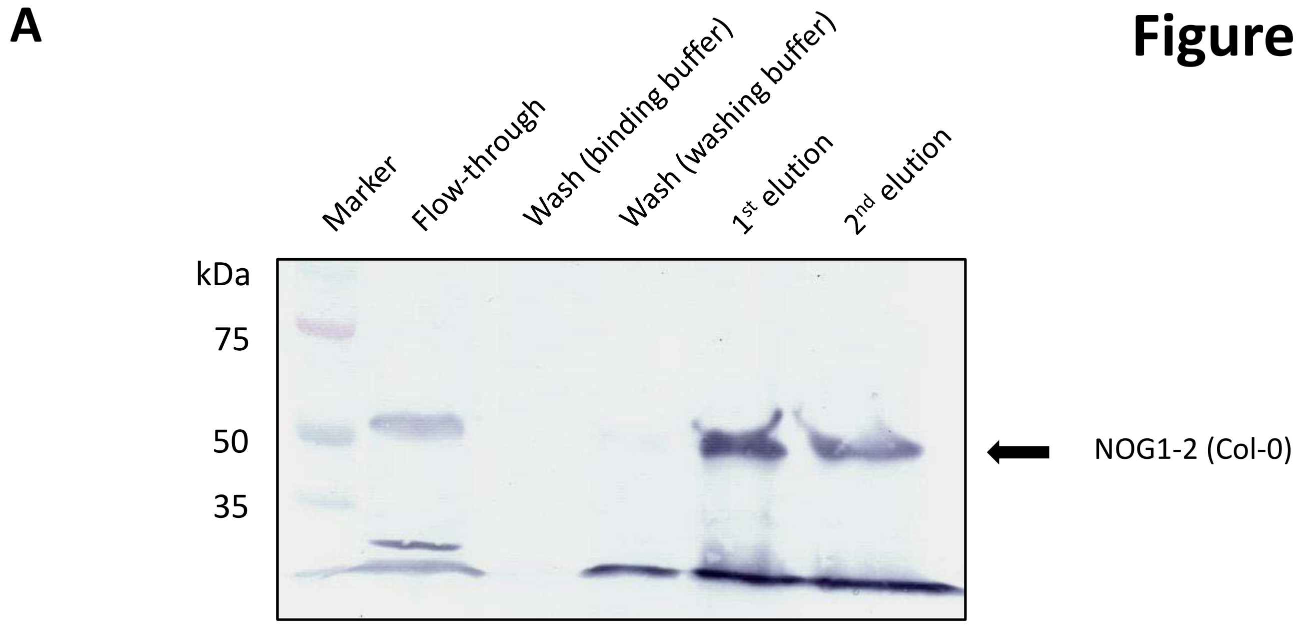

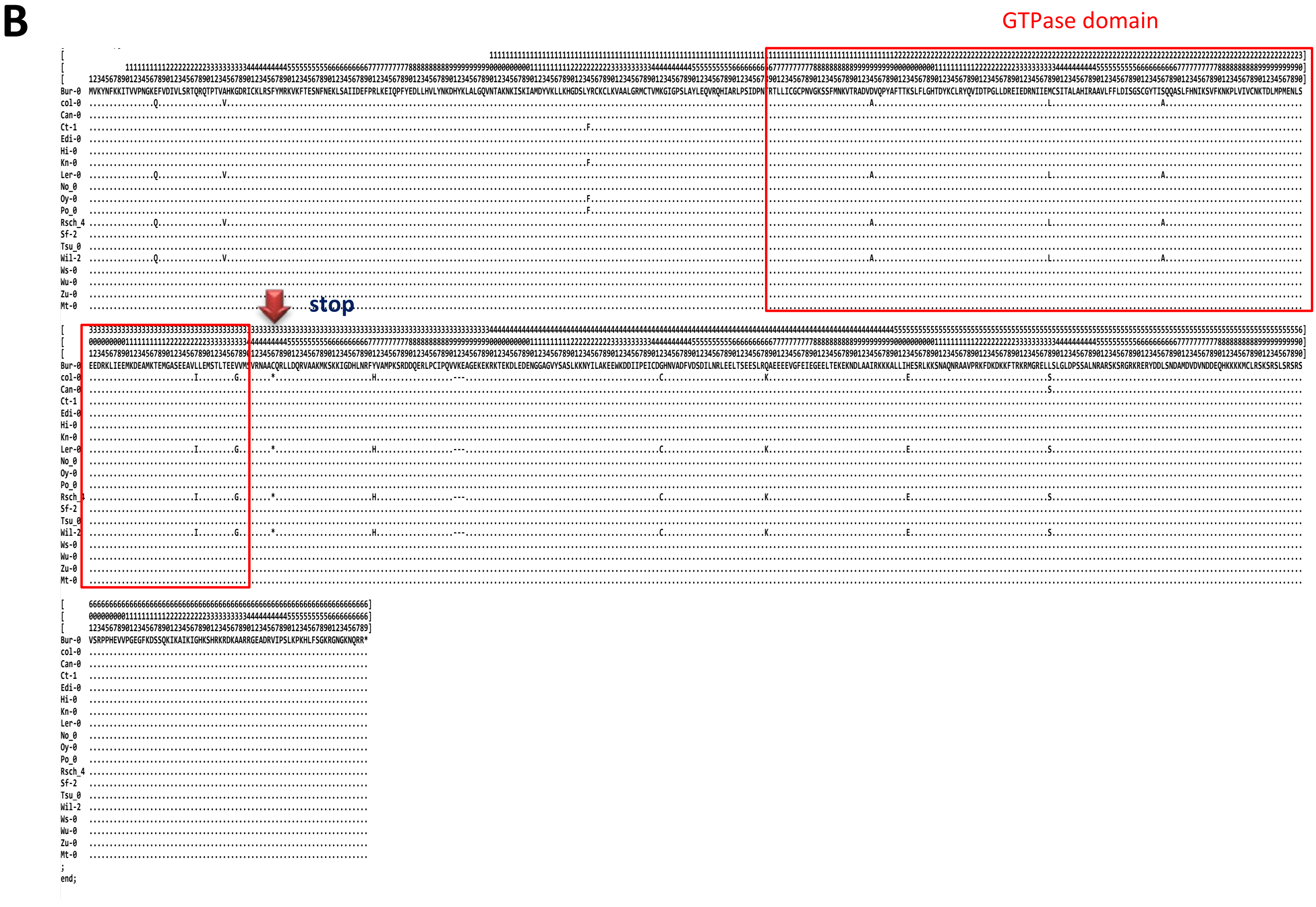

Figure S3. Amino acid sequence alignment of NOG1-1 and NOG1-2 among different Arabidopsis ecotypes, and expression of NOG1-2 protein *in vitro*.

(A and B) Early termination of NOG1-2 expression (truncated form of AtNOG1-2) in Col-0. The full length recombinant NOG1-2 (annotated Arabidopsis database, www.arabidopsis.org) was expressed in *E. coli*. Full lengths of Arabidopsis NOG1-2 cDNA were cloned into the pET59 vector (Novagen) to produce N-terminal His-tagged fusion protein. The plasmid was transformed into Rosetta *E. coli* strain (Novagen). Bacterial cells were grown in LB medium with 50µg/ml carbenicillin to a density of OD_{600}=0.4-0.6. Expression of recombinant proteins was induced overnight at 19ºC with 0.2mM IPTG. Proteins were extracted by using CelLytic B cell lysis buffer (Sigma-Aldrich) and purified using Ni-NTA agarose (Qiagen). The expression of NOG1-2 protein was confirmed by western blot using 6xHis antibody (A). The early termination of NOG1-2 is found in four ecotypes, Col-0, Ler-0, Rsch-4 and Wil-2 (B).

Figure S4

pAtNOG1-1-GUS

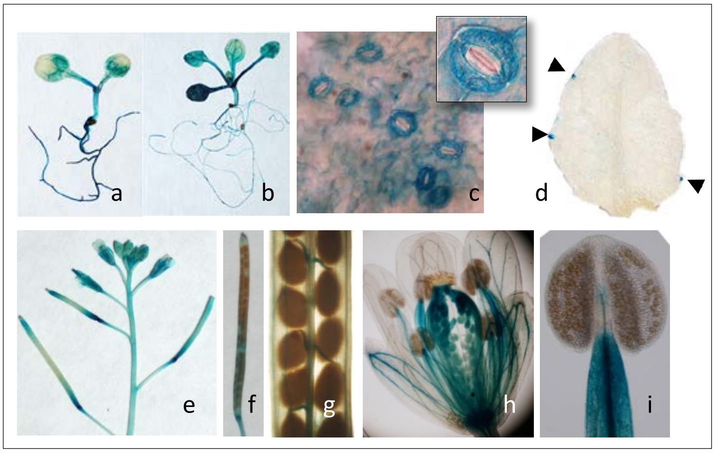

pAtNOG1-2-GUS

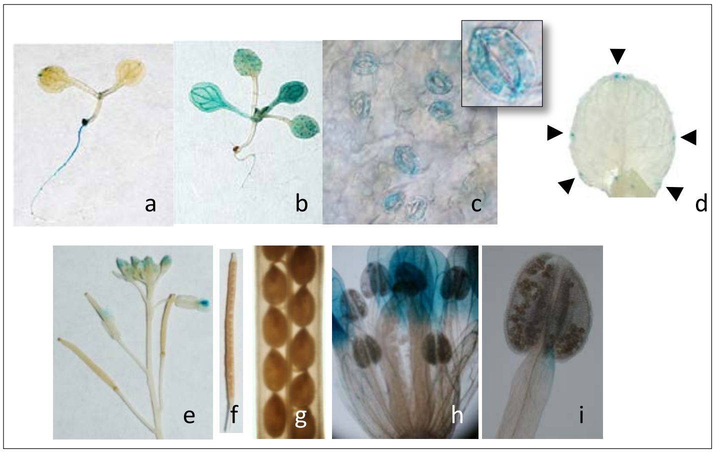

Figure S4. *NOG1-1* and *NOG1-2* promoter-GUS gene expression in different tissues of transgenic Arabidopsis. β-Glucuronidase (GUS) expression driven by *AtNOG1-1* and *AtNOG1-2* promoters in: one-week-old (a) and two-week old (b) seedlings expressing either *AtNOG1-1* or *AtNOG1-2* promoter fusions to GUS, grown on 1x MS medium. GUS expression was seen in guard cells (c) and hydathodes (d). GUS expression was also seen in floral parts (e, h), nectarines at the base of an early developing silique (g) and throughout a maturing silique (f) and anther (i). The T2 lines were used for total three independent experiment. Each experiment had 10 replications and showed similar results.

Figure S5

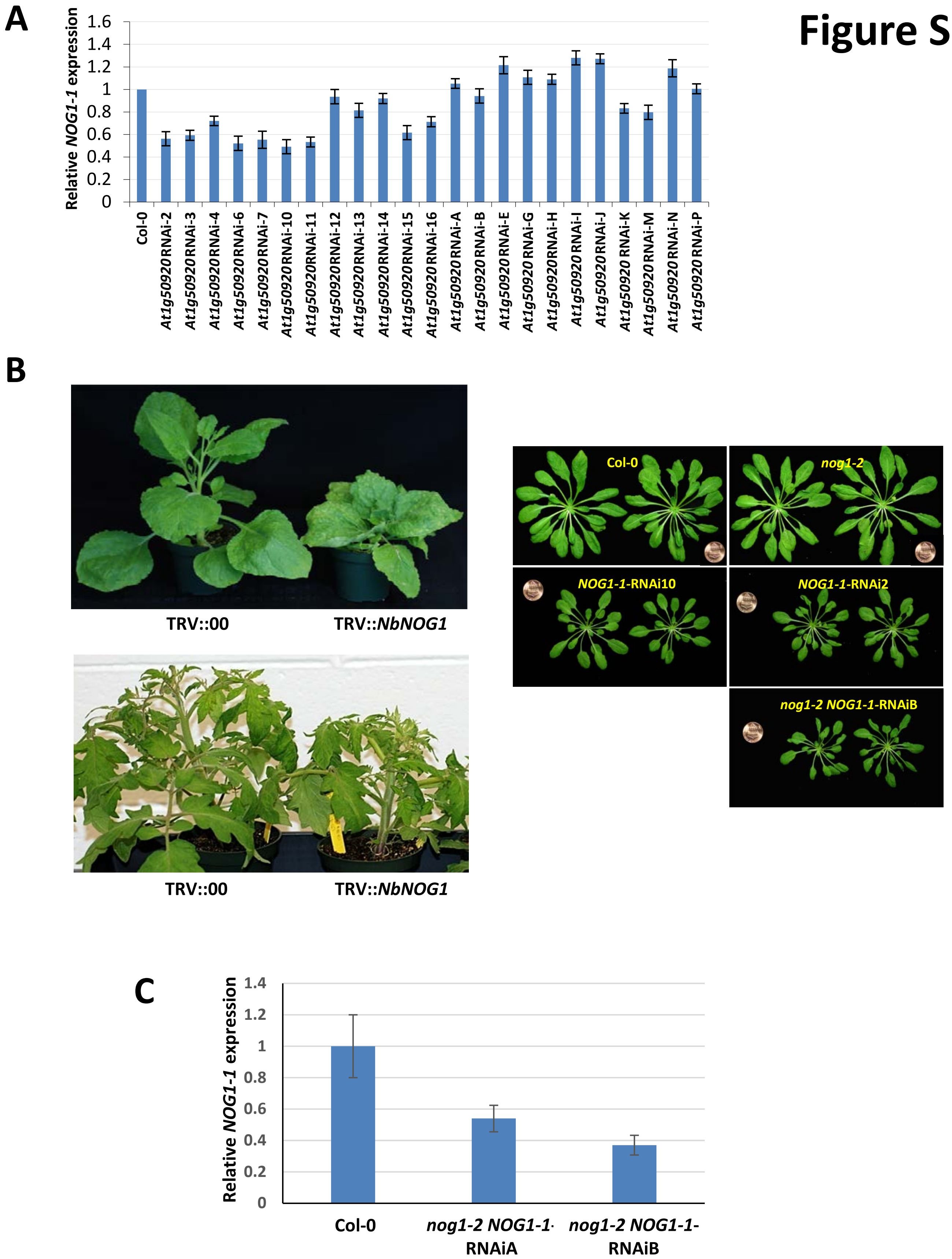

Figure S5. Down-regulation of *NOG1-1* (RNAi) and *NOG1-2* (*nog1-2*; T-DNA mutant), and phenotypes of *NOG1* silenced/mutant plants. (A) Wild-type Col-0 and 23 T1 plants containing the *NOG1-1* RNAi transgene were tested for expression of *NOG1-1* by qRT-PCR. The population of *NOG1-1*-RNAi transgenic lines were developed twice (1st; numeric order, and 2nd; alphabetic order) to obtain the most down-regulation of *NOG1-1*. (B) Mutation/downregulation of *NOG1-2* and *NOG1-1* inhibit plant development.
(C) Down-regulation of *NOG1-1* was determined in two double mutant mimics, *nog1-2 NOG1-1*-RNAiA and *nog1-2 NOG1-1*-RNAiB. *AtUBQ5* was used as an internal control.

Figure S6

A

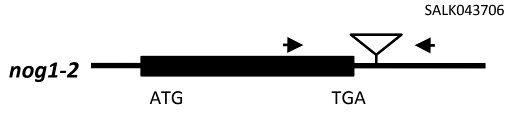

>SALK_043706.41.70.x (sequence before T-DNA insert)
GAAGAAGACGTTAGGTTTTGAGATTGAAGGCGAAGAACTTACAGAGAAGGAGAAGAATGATTTGGCTGCTATTCGTAAGAAGAAAGCT
>SALK_043706.41.70.x (sequence of T-DNA)
GACGCTCAGACCAGTACGAGGCTTCAAGACATGACGCCCGAGCAGACGCCCAAGAGTGCCCAAGAAGAAGACGTTAGGTTTTGAGATTGA
AGGCGAAGAACTTACAGAGAAGGAGAAGAATGATTTGGCTGCTATTCGTAAGAAGAAAGCTGCAGCCCGGGCCGTCGACCACGCGTGCCC
TATAGTGAGTCGTAAAANGCANNGNNTGNNGNGNGNNGGAAGNNGNNGGTGGGGGGGGGGGGGGGGGGGGGGGGGGGGGGGGG
GGGGAGGGGGGGGGGGGGNGGAGGGGGGGGGGGGGAGAGGAGGGGGGAGGGTGGGNGTGGGGGGGGGGGGGGGGGGGAGGGGGG
GGGAAGGGAAGTGGGGGGGGGGGGGAGAGGGGGGGGGGTGATGGGA

B

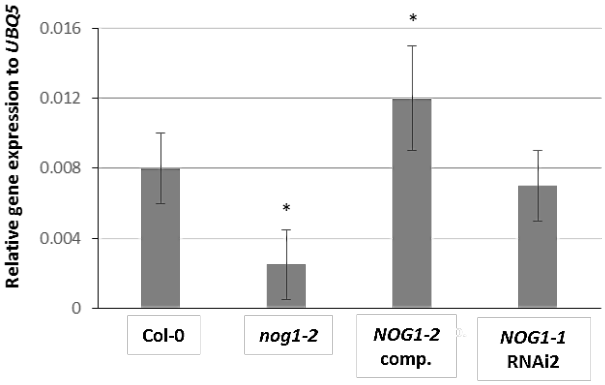

C

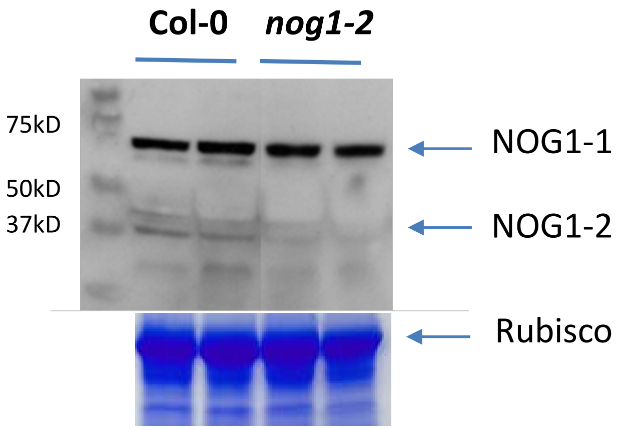

D

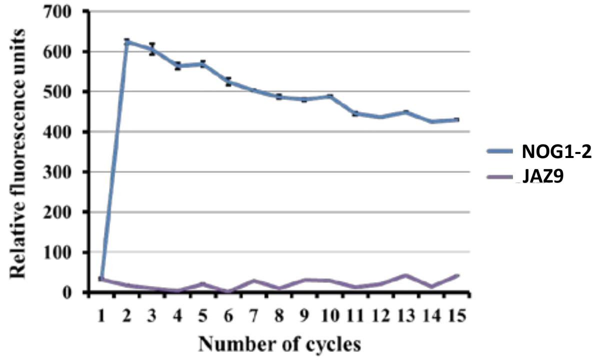

Figure S6. Position of T-DNA insertion in *NOG1-2* and expression of *NOG1-1* in wild-type and mutants.
(A) The position of the T-DNA insertion sites in *nog1-2* line (exon shown as black box). *nog1-2*; SALK043706. The sequence describes the exact location of T-DNA insert at the 3' UTR of *NOG1-2* gene.
(B) Quantitative real-time RT-PCR results showed that *NOG1-2* expression is dramatically reduced in *nog1-2*, but increased in *nog1-2* complemented line (*NOG1-2* comp). *NOG1-2* expression was not altered in *NOG1-1-RNAi*. RNA from two week old seedlings was extracted for cDNA synthesis and RT-PCR. *AtActin2* and *AtUBQ5* was used for internal controls and normalization.
(C) Western blot analysis was performed to determine the NOG1-2 protein in Col-0 and *nog1-2*. Membranes were incubated with anti-GTPBP4 (human) antibodies (N-terminal specific binding). The N-terminal of GTPBP-4 is highly similar to NOG1-2 in plants. Protein expressions of NOG1-2 and NOG1-1 were examined in Col-0 and *nog1-2*. The Rubisco stained with coomassie brilliant blue was shown as loading control.
(D) Fluorescence based assay. First half of the curves (going upwards) represent GTP binding whereas the downwards slope represents GTP-hydrolysis, suggesting the GTPase activity of NOG1-2. JAZ9 protein known stomatal regulation and containing no known GTPase domain was used as the negative control. GTPase activity of NOG1-2 was further confirmed by phosphate release assay as shown in Figure 6A.

Figure S7

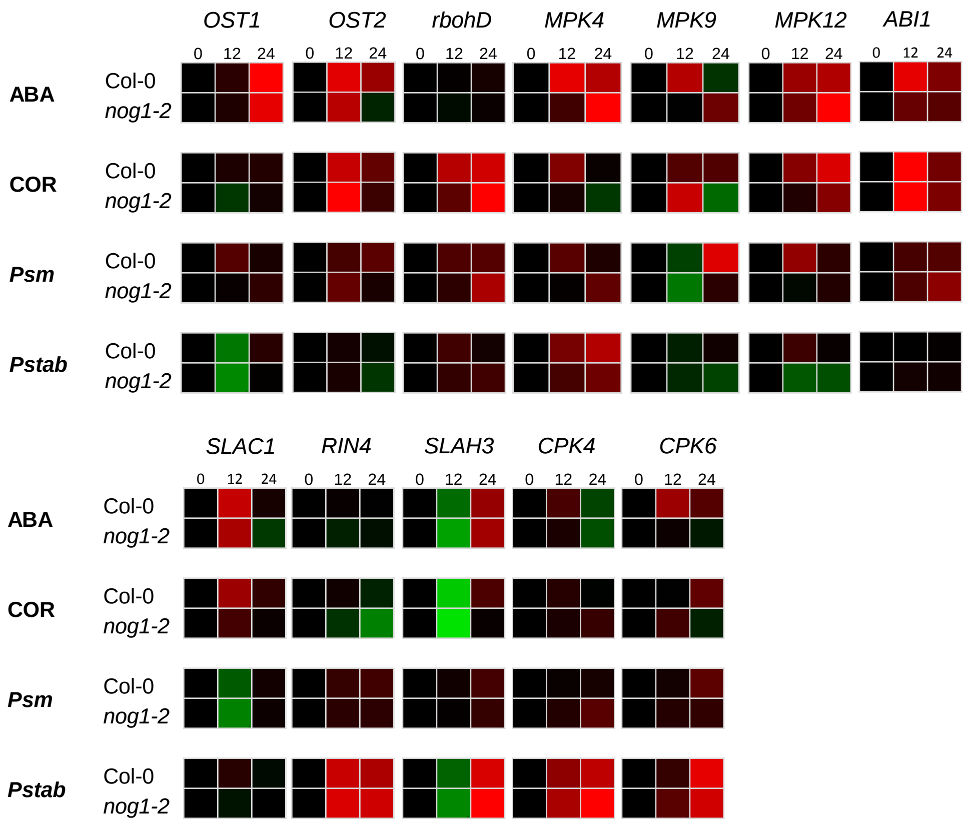

Figure S7. Determination of expression profiling of genes involved in guard cell signaling pathway in Col-0 and *nog1-2* after biotic and abiotic stress treatments. Three weeks old Arabidopsis seedlings grown in MS medium were inoculated with ABA, COR, *P*. *syringae* pv. *maculicola* (*Psm*) and *P*. *syringae* pv. *tabaci* (*Pstab*), and samples were collected at 0 hr, 12 hr, and 24 hr after inoculation for RNA extractions. qRT-PCR analysis was performed with three biological and technical replications. OST1: Open Stomata 1, rbohD: Respiratory Burst Oxidase Homologue D, MPK4: MAP Kinase, ABI1: ABA Insensitive 1, SLAC1: Slow Anion Channel-Associated 1, RIN4: Rpm1 Interaction Protein 4, SLAH3: SLAC1 Homologue 3, CPK4: Calcium-Dependent Protein Kinase 4 .

Supplementary Table S2: Nucleotide sequences from 1100 to 1102 of AtNOG1-2 in various ecotypes

Accession	Origin	AIMS Stock Centre #	Nucleotide from 1100 to 1102 bp
**Bur-0**	Ireland	CS6643	TGT
**Can-0**	Canary Isles	CS6660	TGT
**Ct-1**	Italy	CS6674	TGT
**Edi-0**	Scotland	CS6688	TGT
**Hi-0**	Netherlands	CS6736	TGT
**Kn-0**	Lithuania	CS6762	TGT
**Ler-0**	Poland, formerly Germany	CS20	**TGA**
**Mt-0**	Libya	CS1380	TGT
**No-0**	Germany	CS6805	TGT
**Oy-0**	Norway	CS6824	TGT
**Po-0**	Germany	CS6839	TGT
**Rsch-4**	Russia	CS6850	**TGA**
**Sf-2**	Spain	CS6857	TGT
**Tsu-0**	Japan	CS6874	TGT
**Wil-2**	Russia	CS6889	**TGA**
**Ws-0**	Russia	CS6891	TGT
**Wu-0**	Germany	CS6897	TGT
**Zu-0**	Germany	CS6902	TGT
**Col-0**	Columbia	CS1092	**TGA**

Supplementary Table 4: List of primers used in this study

Primers	Sequences	
31H3-B1	GGGGACAAGTTTGTACAAAAAAGCAGGCTYYATGGTGCAGTATAATTTTAAG	Cloning full length NbNOG1 in N. benthamiana
31H3-B2	GGGGACCACTTTGTACAAGAAAGCTGGGTYCTAGCGCCGGTCAGTTTTCCCAG	
At1g10300-B1(S)	GGGGACAAGTTTGTACAAAAAAGCAGGCTYYATGGTGAAATATAATTTCAAGAAGA	Cloning full length AtNOG1-2 in Arabidopsis
At1g10300-B2(S)	GGGGACCACTTTGTACAAGAAAGCTGGGTYTCAACGCCTTTGGTTTTTTCCA	
At1g50920-B1	GGGGACAAGTTTGTACAAAAAAGCAGGCTYYATGGTTCAATATAATTTCAAG	Cloning full length AtNOG1-1 in Arabidopsis
At1g50920-B2	GGGGACCACTTTGTACAAGAAAGCTGGGTYTCAACGCCTGTCGGTTTTTC	
**Primers used for *NOG1-2* and *NOG1-1* gene expression in Arabidopsis**		
At1g10300RT-F	GTCACTAAGTGTATCAATTTCGTGCAG	NOG1-2 expression, semi RT-PCR
At1g10300RT-R	ACAGTACACATACGCCCAAGAGCA	
At1g50920qRT-F2	GCTGACAGAGTTATACCAACGCTTAGACCG	NOG1-1 expression, real time PCR
At1g50920qRT-R2	CTCTTGTTTGGTTTCATCAACGCCTGTCGG	
At1g50920RT-F(3)	GTGCCTGGTGAAGGATTCAAAGAC	NOG1-1 expression, real time PCR
At1g50920RT-R(3)	CAGAGATTCTTCAACGGATCCAGC	
At1g10300RT-F(3)	AGCAAGTCTAGAGGCAGAAAGAGG	NOG1-2 expression, real time PCR
At1g10300RT-R(3)	TGAGCCCTTGTTACCTGATCACTG	
10300qRT-F	GTCGTGGAGAAGCAGACAGAGTTA	NOG1-2 expression, real time PCR
10300qRT-R	AGCCCTTGTTACCTGATCACTGGT	
At1g10300qRT-F2	CACGTCGTGGAGAAGCAGACAGAGTTATAC	NOG1-2 expression, real time PCR
At1g10300qRT-R2	GCATGGCTTATACTAATGAGCCCTTGTTACC	
AtEF1a-qRTf	TTCACCCTTGGTGTCAAGCA	Internal control for real time PCR
AtEF1a-qRTr	TTTCATCGTACCTGGCCTTGGA	
AtUBQ5 qRTf	GCCGAAGAAGATCAAGCACAAGCA	Internal control for real time PCR
AtUBQ5 qRTr	ACTCCTTCCTCAAACGCTGAACCT	
AT1G10300gusGW-[illegible]	GGGGACAAGTTTGTACAAAAAAGCAGGCTTCGTCGTATGCGGTAGTACGTAGTTG	NOG1-2 GUS expression in Arabidopsis
AT1G10300gusGW-[illegible]	GGGGACCACTTTGTACAAGAAAGCTGGGTTGTAGAGACGATGGTCATTTGGTCC	
AT1G50920gusGW-[illegible]	GGGGACAAGTTTGTACAAAAAAGCAGGCTTCAGAGACCTGCAACTGCAATA	NOG1-1 GUS expression in Arabidopsis
AT1G50920gusGW-[illegible]	GGGGACCACTTTGTACAAGAAAGCTGGGTTTGGGAACAACTGTGATCCTCTTGA	
AT1G10300gfp-R	GGGGACCACTTTGTACAAGAAAGCTGGGTTACGCCTTTGGTTTTTTCCA	NOG1-2 GFP expression in Arabidopsis

AT1G50920gfp-R	GGGGACCACTTTGTACAAGAAAGCTGGGTTACGCCTGTCGGTTTTTC	
10300C-F	GGGGACAAGTTTGTACAAAAAAGCAGGCTTCAAACCCTGGAAGCGCATTGTGT	NOG1-2 complementation in Arabidopsis
10300C-R	GGGGACCACTTTGTACAAGAAAGCTGGGTTAAGCAACGTAACCGACCCAGATTC	

Primers used for gene expression profiling for hormonal defense and guard cell signaling

AtPLDalpha-qrtF	GCACGCCGTTTCATGATTTAC
AtPLDalpha-qrtR	GCTAACATCAACCAGAGGTCAA
AtABI1-qrtF	CGCAGGAGGGAAAGTGATT
AtABI1-qrtR	CTTGAAACCATCCATCATTCCTG
AtBIK1-qrtF	GCGATCCCGTCAAAGTGATA
AtBIK1-qrtR	TTGGACTAGCTAGAGACGGT
AtABF3-qrtF	TGGAGAAAGTGATTGAGAGAAGG
AtABF3-qrtR	GAACTGGAAGCAGAAATTGCG
AtBAK1-qrtF	TCTTTGATGTACCAGCTGAAGAG
AtBAK1-qrtR	TGAACTACAAGTTGCTTCGGAT
AtFLS2-qrtF	CGAAGATGGAAGCACCACA
AtFLS2-qrtR	TCCAGAGTTTGCTTATATGAGGAA
AtBRI1-qrtF	GTTCGATTCCTGATGAGGTAGG
AtBRI1-qrtR	CTCAGGCTATGTCAGCTCTTAC
SALK JAZ9 LP	TCATGCTCATTGCATTAGTCG
SALK JAZ9 RP	AGGGTTAAGTACGAAGGCAGC
AGB1-qrtF	GAGGACACAGGAGAGTGATTTG
AGB1-qrtR	TCCACAAACCGAACCTTACTT
AGG1-qrtF	GAAGGACCAAATGGAGGAGAA
AGG1-qrtR	CCTAGCAAGAAACTAGTATATGTAACAC
AGG2-qrtF	TGAATGCGACATGGGATCAA
AGG2-qrtR	TTTGAGAGAAGGAAAGGAAGATCA
RGS1-qrtF	GATGCAGTAGTCCCGGTTAAG
RGS1-qrtR	GAAGGAAGATTACATGGATTGGATTG
ABI1-qrtF	AGAGAGGAAGCAAAGACAACAT
ABI1-qrtR	ACCCTCTCTGCCTCAGTT
FLS2-qrtF	CGTAACGAGGATCGAGAAGTTT
FLS2-qrtR	AGTATTCAACCTTCGTAACAGAGT
AtPLDalpha-qrtF	TACCTGCCTCCAATCCTTACA

AtPLDalpha-qrtR	CAAAGCTACAACAGCAGCAAAG
AtSLAC1-qrtf	TTTGAAGCAGAGGAAGAGTC
AtSLAC1-qrtr	CATACAATATGCTATCTCACCTACT
AtSLAH3-qrtf	TCCGACAGCAGTCAGAGTAA
AtSLAH3-qrtr	TCGGATACGACTCTGTATTAGGG
AtAHA1/AtOST2-qrt	CGCAAAGCTAAAGGGATTGG
AtAHA1/AtOST2-qrt	CGGTAAATGTTTGTGTTGTTGTG
AtRIN4-qrtf	CGTGAAGAGAGAAGTTCTGGAG
AtRIN4-qrtr	CAAAGCAGCAACATGAGGAAG
AtrbohD-qrtf	CTACTGTGGAATGCCAGGAA
AtrbohD-qrtr	GAAGTTCTCTTTGTGGAAGTCAAA
AtCPK6-qrtf	AAATGCTGGTGTAGGGAGAAG
AtCPK6-qrtr	ATTTGCATCCTCACCGAATAGA
AtCPK4-qrtf	CACAAGCAGCACTGCTAAATC
AtCPK4-qrtr	TTGTGAGTTCTCATTTCTCAATTCC
AtAHK5-qrtf	GCAAATGGTATGGACTCGTTTATT
AtAHK5-qrtr	ATCTGAAATCTCAGTGCAAATACTG
AtSID2-qrtf	ACAGGGATAGTAGCTGGAAGT
AtSID2-qrtr	CTGTAGAGATGTTGTTGCTTCATATTC
